# A predictive descriptor for the d-band center in intermetallic alloys accelerates the design of robust molecular switches

**DOI:** 10.1039/d5sc08297h

**Published:** 2026-01-13

**Authors:** Sha Yang, Junjun Zhou, Yirong Zhang, Guolin Cao, Ji-Chang Ren, Wei Liu

**Affiliations:** a Nano and Heterogeneous Materials Center, School of Materials Science and Engineering, Nanjing University of Science and Technology Nanjing 210094 Jiangsu China syang@njust.edu.cn; b State Key Laboratory of Rare Earth Resource Utilization, Changchun Institute of Applied Chemistry, Chinese Academy of Sciences Changchun 130022 China weiliu@ciac.ac.cn

## Abstract

Breaking the trade-off between stability and switching functionality remains a pivotal challenge in substrate-supported molecular switches. Herein, we propose a design strategy using A_3_B-type intermetallic alloys as substrates to realize a hybrid-bonding precursor state that concurrently achieves robust interfacial stability and enhanced current-switching ratios. We demonstrate that this bistability can be directly predicted from the atomic covalent radius and d-band centers of surface metals. Remarkably, we uncover a distinctive V-shaped relationship between the d-band center of the host metal and valence electron number of the guest metal, governed by the occupancy of d–d anti-bonding states. Furthermore, we elucidate the essential role of geometric and quantum primogenic effects in modulating d–d orbital interactions, resolving longstanding controversies regarding d-band modulation mechanisms for alloys. By incorporating intrinsic parameters, including the valence electron number, atomic radius, and orbital radius of guest metals, we develop a generalizable descriptor for accurately predicting the d-band center of host metals (*R*^2^ > 0.90). This work not only accelerates the exploration of robust room-temperature molecular switches, but also establishes a rational design framework for high-performance intermetallic substrates with optimal adsorption properties, thereby significantly reducing reliance on costly density functional theory calculations.

## Abstract 2

A descriptor derived from elemental parameters accurately predicts the d-band center of host metals on bimetallic surfaces, facilitating the design of robust molecular switches with high interfacial stability and switching performance.

## Graphical abstract

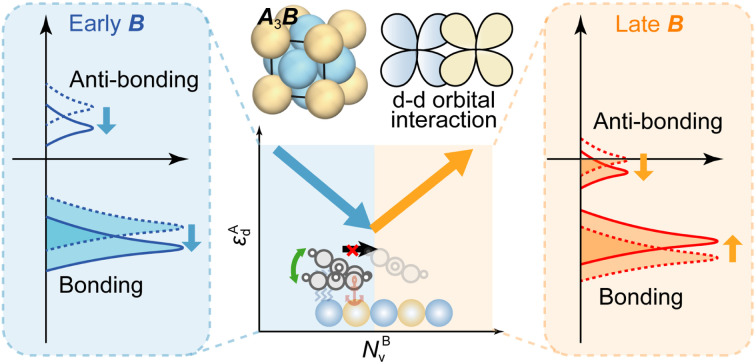

## Introduction

Molecular adsorption on solid surfaces constitutes a fundamental process in chemistry and materials science. While conventional studies often focus on static adsorption configurations, a particularly intriguing functionality arises from reversible dynamic chemical and physical events of the adsorbate.^1,2^ This reversibility forms the basis of molecular switches, showing fascinating opportunities in next-generation memory devices and logic gates.^3–5^ Prototypes include, but are not limited to, azobenzene,^6,7^ diarylethene,^8^ porphyrin,^9,10^ and spin-crossover complexes^11^ on metallic substrates, relying on intrinsic bistability in molecular conformation or spin states. However, a critical challenge remains in balancing interfacial stability with switching functionality.^2^ Excessive electronic coupling may quench switching ability or induce molecular dissociation,^12^ whereas overly weak coupling can lead to thermal diffusion and operational instability.^13,14^ Inspired by the concept of precursor-mediated adsorption,^15^ the reversibility can also be generated by controlling molecule-substrate coupling, particularly the chemisorption and chemisorption precursor state.^16,17^ Representative examples include arene derivatives on metal surfaces^18,19^ and CO on metal tips.^20,21^ However, in spite of the robust structure at the chemisorbed state, the high lateral mobility of molecules at the precursor state often compromises operational reliability.^22^

**Fig. 1:**
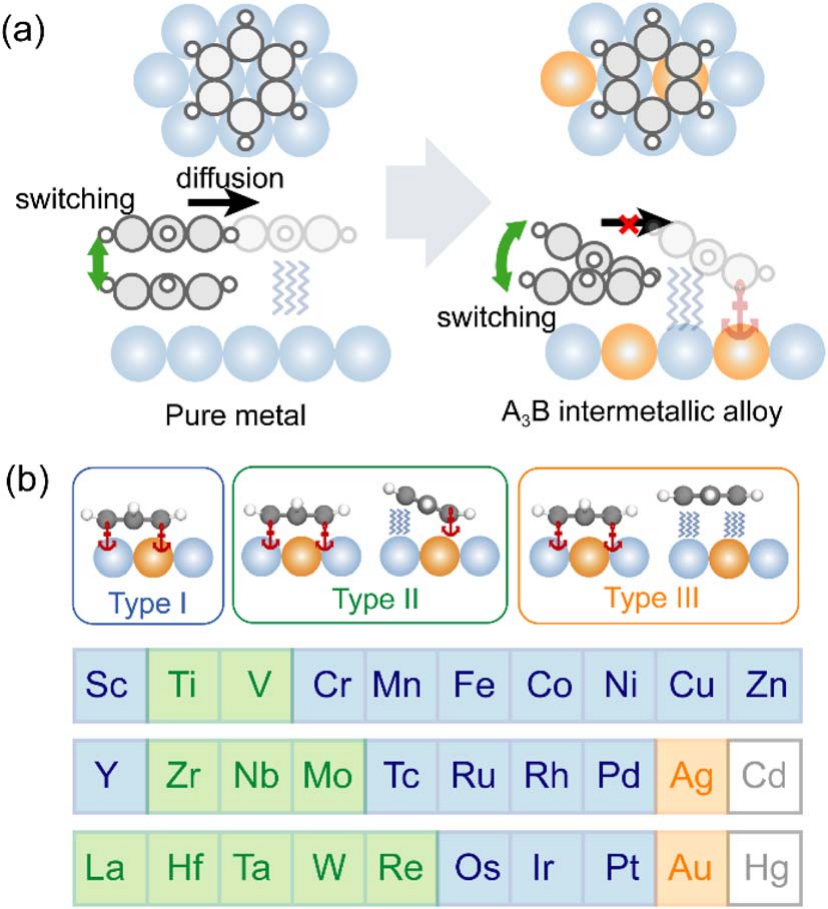
(a) Schematic illustration of the conventional and hybrid-bonding chemisorption precursor states on pure metal and intermetallic alloy, respectively. (b) Three types of adsorption regimes of benzene on Pt_3_M(111), where M represents elements in groups 3–12, except for Cd and Hg. Blue, green, and orange colors represent adsorption type I, type II, and type III.

This mobility originates from the flat potential energy landscape of conventional precursor states, which are characterized by planar physisorbed configurations through long-range van der Waals (vdW) interactions,^17,23^ as illustrated in Fig. 1a. A promising solution to suppress the lateral diffusion is the use of heterogeneous surfaces, such as bimetallic alloys that possess distinct active and noble sites.^24,25^ This can enable a precursor state with coexisting covalent and vdW interactions, thereby ensuring both structural robustness and distinct electronic properties between bistable states.^22,26^ Intermetallic alloys, especially those composed of d-block metals, are ideal platforms for realizing such hybrid-bonding precursor states due to their tunable d-band properties, high stability and ordered structures.^27,28^ Since the adsorbate-substrate bonding scenario is largely governed by the d-band center of surface atoms,^29^ a thorough understanding of its modulation mechanism is essential. However, a consensus remains elusive, owing to the complex interplay of charge transfer,^30^ lattice distortion,^31^ and orbital hybridization.^32^ For instance, while interatomic charge transfer effectively explains the d-band downshift of Pt after alloying with Fe or Ni,^30,33^ the introduction of elements with smaller electronegativity such as Y and Ag leads to an opposite trend.^34,35^ Furthermore, some studies emphasize the crucial role of strain effects in modulating the adsorption activity of Pt surfaces alloying with 3d metals.^27,28^ In contrast, other work has demonstrated that the d-band modulation with late transition metals is dominated by the hybridization between the valence orbitals of adjacent metals, with strain effects being negligible.^36^ Despite these insights, a unified and quantitative predictive framework for the d-band center, which is crucial for the rational design of hybrid-bonding precursor states, is still lacking. Furthermore, while conventional d-band theory has been successful in rationalizing linear scaling relationships for adsorption energies of small adsorbates on transition-metal surfaces, its predictive power is less suited to describing complex potential energy landscapes required for multi-state or switchable interfacial functionalities.

In this work, we introduce a distinct application of d-band modulation aimed at the rational design of a bistable adsorption regime on A_3_B-type intermetallic alloys that simultaneously ensures strong coupling with the substrate and switching functionality (Fig. 1a). Combining feature importance analysis and the sure independence screening and sparsifying operator (SISSO) method,^37^ we extract the key electronic and geometric parameters in governing the adsorption regimes, including the d-band center of the host metal and covalent radius of the guest metal. This enables the construction of a robust classification model for screening bistable systems of benzene on various A_3_B alloy surfaces. We achieve complete discrimination between monostable and bistable adsorption regimes with the investigated dataset (108 systems), while traditional d-band models generally show mean absolute errors around ±0.15 eV in predicting adsorption energies.^38^ By capturing the geometric and quantum primogenic effect, we establish two well-defined inverse linear relationships between the host metal's d-band center and the valence-derived descriptor, validated across diverse intermetallic alloy systems (Pt_3_M, Pd_3_M, Rh_3_M, Ir_3_M). Finally, the robust stability and switching functionality are further confirmed *via ab initio* molecular dynamics (AIMD) and nonequilibrium Green's function (NEGF) transport calculations.

## Results and discussion

### Adsorption regime of benzene on Pt_3_M surfaces

The adsorption behaviors of benzene on Pt_3_M intermetallic alloys were first investigated using the optB88-vdW functional.^39^ The close-packed (111) surfaces of Pt_3_M with *Pm*3̄*m* crystal structure were selected as the substrates because their hexagonal symmetry matches well with that of benzene.^28^ In addition, the potential energy landscape of benzene on Pt(111) shows a shallow minimum near typical physisorption heights,^40,41^ making the benzene/Pt_3_M system suitable for investigating the alloying effect on precursor-mediated adsorption. In particular, eight distinct initial geometries were used to identify the most stable adsorption site (see the Methods section and Fig. S1 for details). We examined 27 Pt_3_M(111) surfaces, where the guest metal M represents elements from groups 3–12, except for Cd and Hg. These intermetallic alloys have been either prepared experimentally or predicted theoretically in previous studies.^27,28,35^

As shown in Fig. 1b, three distinct adsorption regimes were identified, including the monostable interface with a solely chemisorbed state (type I), the bistable interface with a chemisorbed state and a tilted precursor state (type II), and the bistable interface with a chemisorbed state and a planar precursor state (type III). The formation of these adsorption regimes results from a balance between covalent and vdW interactions (Table S1). In all cases, the chemisorbed benzene ring forms covalent bonds with underlying Pt and guest metal atoms. However, the precursor states in types II and III exhibit different bonding scenarios: the precursor state in type III is characterized by a typical physisorption dominated by vdW interactions, whereas in type II, one side of the benzene ring is covalently bonded to the M atom while the other interacts weakly with Pt atoms. As a consequence, the C–M bond in type II acts as an anchor that inhibits the lateral diffusion of the molecule in both states.^42^ The type-II adsorption was further checked by two additional vdW-inclusive methods, the so-called PBE-D3 (ref. 43) and PBE + MBD-nl^44^ (Fig. S2). Notably, type-II adsorption is observed in a series of Pt_3_M systems (M = Ti, V, Zr, Nb, Mo, La, Hf, Ta, W, and Re), demonstrating the generality of this hybrid-bonding precursor state (Fig. S3, S4 and Table S1). Interestingly, guest metals facilitating type-II adsorption are mainly located near group 5, suggesting a non-monotonic modulation effect of the guest metal across the period.

**Fig. 2:**
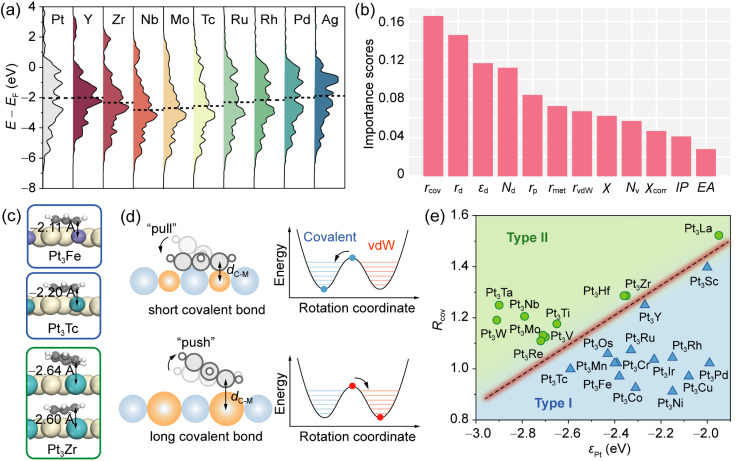
(a) PDOS on the d-band of the surface Pt atom for Pt_3_M alloys. The leftmost panel shows PDOS of the pure Pt(111) surface. The dashed lines represent the d-band center positions of surface Pt atoms. The energy scale is referenced to the Fermi level (*E*_F_ = 0). (b) Feature importance scores of various parameters for the classification of adsorption regimes of benzene on Pt_3_M(111) by a random forest method. (c) Side views of adsorption structures of benzene on Pt_3_Fe, Pt_3_Tc, and Pt_3_Zr. The values represent bond lengths between carbon atoms in benzene and below guest metals. (d) Schematic illustration of how the length of the C–M covalent bond (*d*_C–M_) influences the adsorption regimes. (e) Classification model for prediction of adsorption regimes on various Pt_3_M(111) surfaces using d-band centers of the Pt atom and the ratio of covalent radii between the guest metal and Pt.

To elucidate this trend, we analyzed the d-band characteristics of surface atoms, which play a key role in determining the bonding scenario between the adsorbate and the substrate.^42^ As shown in Fig. 2a, the projected density of states (PDOS) on the Pt d-band in alloys with 4d metals shows varied broadenings and shifts. Notably, as the guest metal moves from left to right in the period, the trend of d-band center shift of the Pt atom (*ε*_Pt_) is not monotonic, reaching a minimum for Pt_3_Nb (−2.79 eV). This downshift lowers the energy location of anti-bonding states between Pt and benzene, increasing their occupancy below the Fermi level, as supported by the crystal orbital Hamilton population (COHP) analysis (Fig. S5). Consequently, the Pt-benzene covalent interactions are weakened, generating two local energy minima above Pt sites. In contrast, an upshift of *ε*_Pt_, as observed in Pt_3_Y (−2.00 eV) and Pt_3_Pd (−1.99 eV), strengthens covalent bonding with benzene, resulting in a single deep energy minimum (chemisorbed state). Since both d-band centers of Y and Zr are significantly higher than *ε*_Pt_, covalent bonding is maintained between benzene and the underlying guest metal atoms. In the case of Pt_3_Ag, despite a decreased *ε*_Pt_, the noble nature of Ag leads to weak adsorption affinity to benzene, yielding a conventional precursor state. Thus, the adsorption regimes of benzene on Pt_3_Y, Pt_3_Zr, and Pt_3_Ag are characterized by types I, II, and III, respectively. These results underscore the essential role of the modulated d-bands of both Pt and guest metals in determining the adsorption behavior of benzene.

While the electronic structure of the host metal is a primary factor, it is not the sole determinant of the adsorption regime. For instance, although Pt_3_Tc presents a lower *ε*_Pt_ than Pt_3_Zr, benzene on Pt_3_Tc(111) forms only a monostable chemisorbed state. Similar deviations are observed in Pt_3_Cr, Pt_3_Mn, and Pt_3_Fe. To quantitatively evaluate the influence of guest metal properties, we performed feature importance analysis using a random forest classifier,^45^ which is well-suited for small datasets. We considered multiple electronic and geometric parameters of the guest metals, including electron affinity (EA), ionic potential (IP), Pauli electronegativity (*χ*), a corrected electronegativity (*χ*_corr_), d-band filling (*N*_d_), valence electron number (*N*_v_), metallic radius (*r*_met_), covalent radius (*r*_cov_), vdW radius (*r*_vdw_), and p- and d-orbital radii (*r*_p_, *r*_d_) (Table S2). Pt_3_Ag and Pt_3_Au were excluded from the analysis due to the noble nature of Ag and Au. As shown in Fig. 2b, the covalent radius of the guest metal was identified as the most important geometric descriptor, aligning with the permutation importance analysis (Fig. S6). This implies that covalent bonding between benzene and the guest metal strongly perturbs the bonding scenario with Pt atoms. For example, guest metals with smaller covalent radii (*e.g.*, Fe and Tc) form shorter C–M bonds, pulling the opposite side of the benzene ring closer to Pt and promoting the formation of covalent bonds (Fig. 2c and d). In contrast, larger *r*_cov_ values (*e.g.*, Zr) allow the benzene ring to be pushed into a vdW-dominated potential well above Pt sites, facilitating the formation of a bistable type-II regime.

### Classification model for monostable and bistable adsorption

Based on the five most important features from the above analysis, we employed the SISSO method^37^ to construct a classification model using the d-band center of Pt and the normalized covalent radius *R*_cov_ = *r*_cov(M)_/*r*_cov(Pt)_. As illustrated in Fig. 2e, clear separation between type-I and type-II adsorption regimes is achieved by plotting *ε*_Pt_ against *R*_cov_. Systems in the top-left region correspond to type-II adsorption, while those in the bottom-right region belong to type I (blue region). This indicates that the hybrid-bonding chemisorption precursor state requires both a downshifted *ε*_Pt_ and a large *r*_cov(M)_ to passivate the adsorption affinity of Pt. To rigorously test the sensitivity of our findings to the choice of exchange-correlation functional, we performed additional calculations using the strongly constrained and appropriately normed (SCAN) *meta*-GGA^46^ combined with the rVV10 dispersion correction.^47^ The classification based on the SCAN + rVV10-calculated *ε*_Pt_ plotted against *R*_cov_ remains fully consistent with the optB88-vdW results (Fig. S7–S9).

**Fig. 3:**
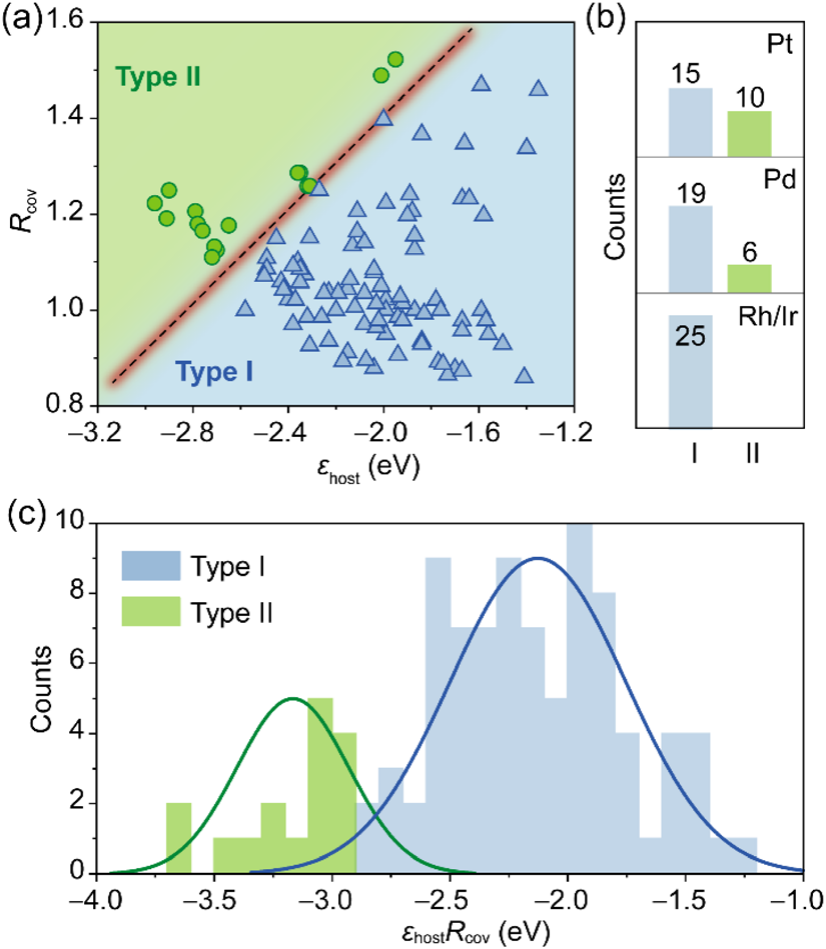
(a) Classification model for prediction of the adsorption regime for benzene on (111) surfaces of Pt_3_M, Pd_3_M, Ir_3_M, and Rh_3_M alloys. The metals M include elements in groups 3–12, except for Ag, Cd, Au, and Hg. (b) Numbers of type-I (light blue) and type-II (green) adsorption regimes for benzene on the (111) surfaces of Pt_3_M, Pd_3_M, Ir_3_M, and Rh_3_M alloys. (c) Classification model for prediction of the adsorption regime using a descriptor of *ε*_host_*R*_cov_.

The classification model shows excellent transferability, achieving 100% accuracy for Pd_3_M, Ir_3_M and Rh_3_M systems (Fig. 3a). Furthermore, we performed leave-one-out cross-validation (LOOCV) on the adsorption regime classification across these systems. Our model (distinguishing between type-I and type-II adsorption) achieves a LOOCV accuracy of 98.15% (106/108 correct), which closely matches the accuracy on the full training set (100%). This minimal gap indicates strong generalization and a low risk of overfitting. The model's excellent discriminative ability is further supported by a receiver operating characteristic (ROC) area under the curve (AUC) value of 99.49%. These results confirm that the descriptors, based on the host metal's d-band center and the guest metal's covalent radius, maintain robust predictive reliability despite the limited sample size.

Notably, the number of type-II systems varies significantly with host metals. For example, Pd_3_M yields six type-II cases (Fig. 3b and S10), while no type-II adsorption is found in Rh_3_M and Ir_3_M systems, likely due to the high intrinsic adsorption affinity of Rh and Ir. A feasible strategy to induce type-II adsorption on Rh- or Ir-based substrates is to enhance the alloying effect by increasing the concentration of the guest metal.^27^ Additionally, through linear combination, we derived a descriptor, *ε*_Pt_*R*_cov_, which captures both geometric and electronic factors, to clearly separate the two adsorption regimes (Fig. 3c and S6).

### Molecular dependence of the classification descriptors

To examine the transferability of the type-II adsorption regime beyond benzene, we performed additional DFT calculations for three representative aromatic molecules, *i.e.*, naphthalene, nitrobenzene, and pyridine, adsorbed on Pt_3_Zr(111) and Pt_3_Nb(111) (Fig. S11). For all three molecules, we consistently observe the coexistence of a conventional chemisorbed configuration and a tilted precursor state, characteristic of the type-II adsorption behavior originally identified for benzene. These results indicate that the descriptor-based classification remains valid for a broader class of aromatic systems whose interfacial interactions are primarily governed by π–d hybridization. Furthermore, these results also suggest practical design strategies for substrate-supported molecular switches. For example, larger π-conjugated frameworks (*e.g.*, naphthalene) may facilitate the formation of well-defined molecular layers and enable finer control over molecular states. In addition, molecular functionalization through heteroatoms (*e.g.*, N, S, or P) can enhance the anchoring effect *via* strong heteroatom–metal interactions, thereby improving overall stability. We note, however, that the absolute adsorption energies, particularly the energy difference between the chemisorbed and precursor states, remain molecule-dependent. Accordingly, while the general design principle of employing early transition metals (*e.g.*, Zr and Nb) in Pt_3_M alloys to induce type-II adsorption appears robust for aromatic hydrocarbons and their derivatives, quantitative predictions for new molecular species require explicit validation. Key parameters, such as the critical d-band center separating type-I and type-II regimes, are therefore expected to be molecule-specific.

### Electronic origin of d-band center shifts on bimetallic surfaces

While covalent radii of elements are readily accessible from the literature,^48^ d-band centers of surface atoms are subtly influenced by adjacent alloying atoms. The mechanism of alloying-induced d-band modulation has long been a topic of interest in molecular electronics^49^ and catalysis,^24^ yet a comprehensive understanding remains elusive. Common hypotheses include interatomic charge transfer, strain effect, and orbital hybridization. We first examined the charge transfer effect but found that the d-band filling of surface Pt atoms remains largely unchanged upon interaction with various d-block metals (Table S3). Moreover, poor correlation is observed between *ε*_Pt_ and charge transfer, as measured against the electronegativity of the guest metal (Fig. S12). A similar analysis of surface Pt–Pt atomic distances across different intermetallic alloys also reveals weak correlation with *ε*_Pt_ (Table S4). Furthermore, orbital hybridization is assessed *via* the d-orbital coupling matrix element;^50^ still, no clear relationship with *ε*_Pt_ emerges (Fig. S12). These results suggest that shifts in the d-band center likely stem from a synergetic combination of multiple factors rather than a single mechanism.

**Fig. 4:**
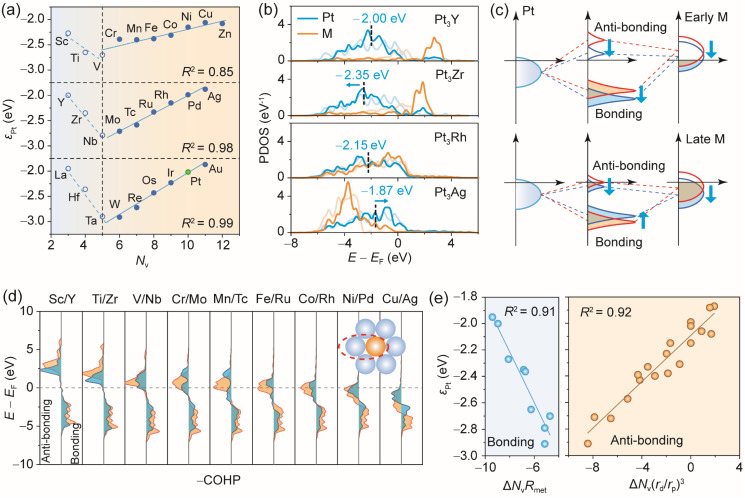
(a) Relationship between the d-band center of surface Pt atoms (*ε*_Pt_) and group number of the guest metal. Green circle denotes the counterpart of pure Pt. (b) PDOS on the surface Pt and M metal atoms in Pt_3_Y, Pt_3_Zr, Pt_3_Rh, and Pt_3_Ag. The shallow-colored lines denote the PDOS of the pure metal. The dashed lines represent the values of *ε*_Pt_. (c) Splitting of bonding and anti-bonding states for d–d orbital hybridization between Pt and early or late d-block metals. (d) COHP for d–d orbital interactions between surface Pt and guest metals M, which include 3d and 4d metals. (e) Linear correlation between the valence electron-derived descriptors and *ε*_Pt_ in Pt_3_M. The energy scale in (b) and (d) is referenced to the Fermi level (*E*_F_ = 0).

To elucidate the underlying modulation mechanisms, we plotted *ε*_Pt_ in Pt_3_M as a function of the guest metal's group number (equivalent to valence electron number *N*_v_) in Fig. 4a. Interestingly, a consistent V-shaped trend is observed across Pt_3_M systems with 3d, 4d, and 5d guest metals. As the guest metal moves from left to right in a given period, *ε*_Pt_ initially shifts downward and then rises, in agreement with previous theoretical studies.^35,36^ Two opposite linear correlations between *ε*_Pt_ and *N*_v_ emerge, separated at group 5. For guest metals beyond group 6, strong linear relationships are observed for 3d (*R*^2^ = 0.85), 4d (*R*^2^ = 0.98), and 5d (*R*^2^ = 0.99) alloy series. To understand this V-shaped trend, we analyzed in detail the d-band of both alloyed atoms and their pure counterparts (Fig. 4b and S13). For example, when Y is replaced by Zr, both the lower and upper edges of the Pt d-band shift downward. In contrast, replacing Rh with Ag causes the upper edge to shift downward while the lower edge remains unchanged, resulting in a narrowing of the Pt d-band. Given that the d-band filling of Pt is nearly constant across these systems, this narrowing leads to an upshift in *ε*_Pt_. These findings indicate the presence of two distinct orbital hybridization mechanisms operative in different regions of the periodic table.

The d–d orbital interactions between Pt and guest metals induce splitting into bonding and anti-bonding states. For early transition metals (*e.g.*, at the beginning of the 4d series), an increase in *N*_v_ corresponds to higher filling and a lower energy position of the d-band. This leads to a downshift of both bonding and anti-bonding states (upper panel of Fig. 4c), resulting in an overall downshift of the Pt d-band. However, this trend reverses once the anti-bonding states begin to occupy below the Fermi level (lower panel of Fig. 4c). As the d-band filling of the guest metal further increases, the d–d orbital interactions give rise to more significant Pauli repulsion. This reduces the covalency between Pt and the guest metal, showing an increased propensity towards charge-shift bonding.^51^ Consequently, the interatomic wavefunction overlap is reduced, which leads to more atomic-like d states^52^ and Pt d-band narrowing. COHP analysis for d–d orbital interactions in 3d- and 4d-alloyed Pt_3_M systems confirms that for the guest metal in groups 3 and 4, all anti-bonding states consistently lie above the Fermi level (Fig. 4d). As *N*_v_ increases, the anti-bonding states gradually shift downward across the Fermi level, leading to a reversal in the trend of *ε*_Pt_. A similar trend is observed in 5d metal-alloyed Pt_3_M systems (Fig. S14). These results demonstrate that the two opposite trends in *ε*_Pt_ as a function of *N*_v_ are substantially governed by the occupancy of d–d anti-bonding states.

Notably, *ε*_Pt_ also exhibits a significant dependence on the period of the guest metal. For early metals in a given group, a strong correlation exists between *ε*_Pt_ and the atomic radius (*r*_met_) of the guest metal. A larger atomic radius introduces tensile strain, reducing wavefunction overlap between adjacent metals. This consequently results in an upshift in *ε*_Pt_, as evidenced in Pt_3_Sc (−2.27 eV), Pt_3_Y (−2.00 eV), and Pt_3_La (−1.95 eV). To capture the strain effect, we introduced a corrected descriptor, Δ*N*_v_*R*_met_, by a linear combination of the normalized valence electron number (Δ*N*_v_ = *N*_v(M)_ – *N*_v(Pt)_) and the atomic radius (*R*_met_ = *r*_met(Pt)_/*r*_met(M)_). This descriptor improves the correlation for Pt_3_M with a guest metal in groups 3–5, increasing *R*^2^ from 0.83 to 0.91 (Fig. 4e and S11). However, this linear relationship breaks down for guest metals beyond group 6, where geometric differences between Pt and the guest metal almost diminish. Interestingly, the correlation slopes differ significantly between Pt_3_M with 3d and 4d/5d guest metals, which we attribute to the quantum primogenic effect in 3d elements.^53^ For 3d metals, nodeless 3d orbitals lack inner-shell d orbitals to enforce orthogonality, causing the valence d orbitals to reside closer to the core and exhibiting similar radial extent to the 3p semi-core orbitals.^54,55^ In contrast, the presence of inner-shell d orbitals in 4d and 5d elements pushes the valence d orbitals to more outer regions than the 4s/4p or 5s/5p orbitals. Consequently, d–d orbital hybridization with Pt is significantly suppressed for 3d metals due to spatial overlap between 3d and 3p orbitals, leading to poor correlation between *ε*_Pt_ and *N*_v_ unless the quantum primogenic effect is considered (Fig. S15).

**Fig. 5:**
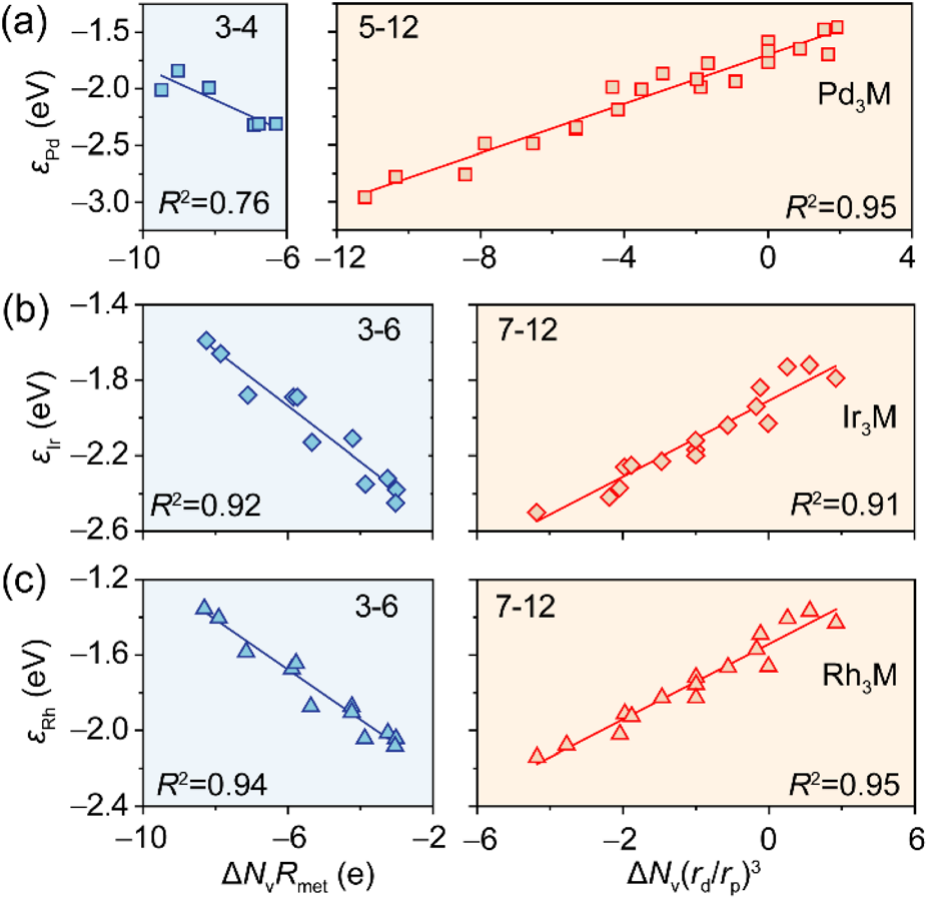
Generalization of the descriptor for predicting the d-band center of the host metal in (a) Pd_3_M, (b) Ir_3_M, and (c) Rh_3_M systems. The group numbers for which the descriptor is applicable are indicated in each panel.

To address this problem, we introduced an orbital volume overlap parameter between the (*n* – 1)p and (*n* – 1)d orbitals of guest metals in the *n*th period, (*r*_d_/*r*_p_)^3^, where *r*_d_ and *r*_p_ represent the orbital radii of d and p orbitals,^56^ respectively. A smaller value of this parameter indicates a more pronounced primogenic effect. Given the minimal geometric influence, we omitted the atomic radius factor and proposed a new descriptor Δ*N*_v_(*r*_d_/*r*_p_)^3^, improving *R*^2^ from 0.78 to 0.92. This descriptor proves generalizable across Pd_3_M, Rh_3_M, and Ir_3_M systems (Fig. 5 and S11). The relatively poor correlation observed for Pd_3_M with group 3–4 guest metals can be attributed to the limited data set (Fig. 5a). Notably, the turning point of the V-shape relationship in the d-band center trend varies with the host metal (Fig. S16). As evidenced by Pd_3_M systems, the higher d-band filling of Pd compared to Pt leads to prominent occupancy of anti-bonding states below the Fermi level with V/Nb/Ta (Fig. S17, S18 and Table S5). As a result, the minima of Pd d-band centers consistently appear at group 5. Conversely, the lower d-band filling of Rh and Ir than Pt causes anti-bonding states to become occupied only with guest metals from group 7 onward (Fig. S19). Therefore, the applicability of the descriptor Δ*N*_v_*R*_met_ is extended to groups 3–6 (Fig. 5b and c). These findings resolve the controversy regarding the d-band modulation mechanism in alloys and provide a predictive model for designing intermetallic alloys with desired electronic properties.

### Molecular switching performance of the type-II adsorption regime

**Fig. 6:**
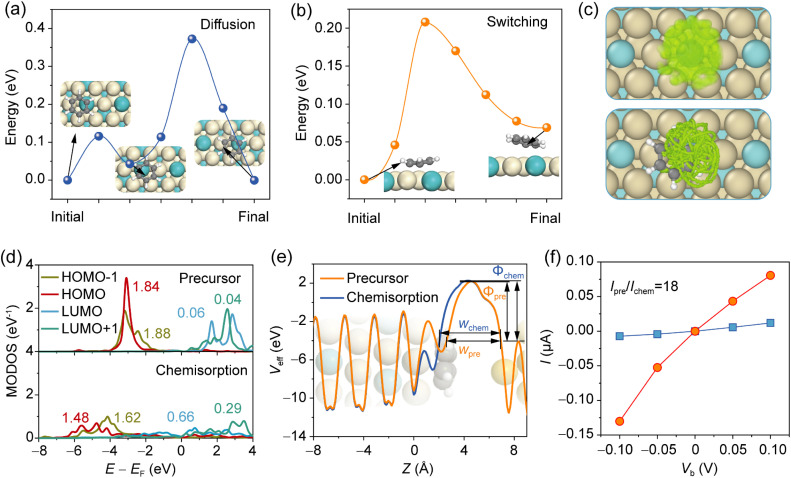
(a) Diffusion of benzene between neighboring precursor sites. (b) Switching pathway from chemisorption to precursor states. (c) Spatial distribution (upper panel) and trajectory (lower panel) of carbon atoms in AIMD simulation of benzene on Pt_3_Zr(111). (d) Molecular orbital density of states (MODOS) of benzene on Pt_3_Zr(111) at the precursor and chemisorption states. (e) Effective potential profile of Pt_3_Zr/benzene/Au contact. (f) Currents at two states under various bias voltages. The value denotes the current ratio under a bias voltage of −0.10 V.

To validate the potential of the type-II regime for molecular memristors, we further investigated the dynamic, electronic, and transport properties. The benzene/Pt_3_Zr system was selected as a representative case due to the comparable stability of its two bistable states and the low tendency for segregation in the first two layers of the Pt_3_Zr surface.^57^ Using the climbing image nudged elastic band (CI-NEB) method, we determined a switching barrier of 0.20 eV between the chemisorbed and precursor states, and a diffusion barrier of 0.38 eV between adjacent precursor sites (Fig. 6a and b). These results confirm that the switching process can occur without significant lateral diffusion. The improved diffusion barriers at the precursor state are also evidenced in Pt_3_Ti (0.40 eV) and Pt_3_Hf (0.37 eV) systems (Fig. S20), which are significantly higher than those reported in previous studies, such as tetrachloropyrazine on Pt(111) (0.12 eV)^17^ and anthradithiophene on Cu(111) (0.20 eV).^42^ In addition, we have also carried out AIMD calculations for 10 ps at 300 K. The spatial distribution and trajectory show that the benzene molecule remains localized above the Zr atom despite rotational motion (Fig. 6c).

These distinct molecular adsorption structures give rise to different electronic and transport properties. As shown in Fig. 6d, the molecular orbital density of states (MODOS) reveals greater shifting and broadening of frontier orbitals in the chemisorption state compared to the precursor state. The differential occupation of these orbitals suggests that the switching process could be induced by applying a suitable bias to inject hot carriers into the molecule.^19^ When integrated into a junction, the difference in interfacial charge redistribution between the two states yields distinct effective potential profiles (see Methods and Fig. S21). Within Landauer formalism,^58^ the carrier tunneling probability is inversely proportional to the value of *Φ*_TB_(*w*_TB_)^2^, where *Φ*_TB_ and *w*_TB_ are the tunneling barrier height and tunneling width, respectively.^59^ The evidently smaller *w*_TB_ at the precursor state would enhance the tunneling probability (Fig. 6e), resulting in distinct transport properties. At the voltage bias of −0.1 V, a current ratio of up to 18 is achieved (Fig. 6f), which is the highest reported for this type of molecular switch.^18,60^ These results confirm the feasibility of using intermetallic alloys as substrates for robust molecular switches.

## Conclusions

We have successfully designed a new type of molecular switch based on benzene adsorbed onto A_3_B intermetallic alloy surfaces, which exhibits robust room-temperature stability and a high current switching ratio. The superior switching performance originates from the unique hybrid covalent-vdW bonding characteristics of the chemisorption precursor state, which are substantially governed by the d-band center and the covalent radius of surface atoms. We unraveled two distinct mechanisms of d–d orbital hybridization in d-block metal alloys, governed by the occupancy of the anti-bonding states. These mechanisms can be predicted directly from the valence electron number, allowing intuitive identification of the direction of d-band center shifts for a given host metal without additional computation. By accounting for the geometric and quantum primogenic effects, we developed a linear regression model for the quantitative prediction of the d-band center of host metals. While this study focuses on d–d orbital hybridization in bimetallic alloys, the developed framework is readily generalizable to more complex alloys by accounting for the nearest–neighbor interactions. Therefore, this work not only advances molecular switch design but also offers a general strategy for the electronic modulation of intermetallic alloys in applications ranging from nanoelectronics to catalysis, which significantly reduces the need for extensive computational screening.

It should be noted that the present model is based on ideal, clean bimetallic surfaces. Under realistic operating or ambient conditions, however, surfaces containing early transition metals such as Zr are prone to oxidation. Previous theoretical studies have shown that oxygen adsorption on Pt_3_Zr is strongly exothermic (Δ*E* ≈ −5 eV), which can promote the formation of ultrathin ZrO_2_ layers and substantially modify the surface electronic structure and adsorption properties.^61,62^ Such oxidation effects may, in turn, influence the switching behavior predicted in this work. To address this limitation, we note that future studies should combine (i) experimental characterization of the surface composition of Pt_3_M alloys under realistic conditions, for example using X-ray photoelectron spectroscopy, with (ii) explicit first-principles modeling of more realistic interfacial structures, such as Pt/ZrO_2_/Pt_3_Zr heterostructures. These efforts will be crucial for quantitatively assessing oxidation effects and for guiding the design of robust surface or interface engineering strategies.

## Methods

All DFT calculations were performed using the Vienna *Ab initio* Simulation Package (VASP).^63^ Structural optimizations and electronic analyses were performed using the optB88-vdW functional^39^ due to its proven accuracy and efficiency in describing the structure and stability of benzene on transition metal surfaces, including Cu(111), Ag(111), and Pt(111), as demonstrated in prior benchmarks.^40^ The type-II adsorption regime has also been examined using the nonlocal many body dispersion (MBD-NL) method^44^ coupled with the Perdew–Burke–Ernzerhof (PBE) functional,^64^ as implemented in the all-electron Fritz Haber Institute *ab initio* molecular simulations (FHI-aims) package,^65^ and using the PBE-D3 (ref. 43) method. The energy convergence criterion was set to 10^−5^ eV, and the structures were optimized until the forces on each atom fell below 0.05 eV Å^−1^. Reducing the force threshold to 0.02 eV Å^−1^ resulted in adsorption energy changes of less than 20 meV. A 5 × 5 × 1 Monkhorst–Pack k-mesh was used for Brillouin zone sampling.^66^ Further increasing the k-mesh to 7 × 7 × 1 resulted in total energy variations of 5 meV, indicating that a 5 × 5 × 1 mesh is enough for the energy calculations. For density of states calculations, the k-mesh was increased to 9 × 9 × 1. To keep consistency, the NBANDS tag in density of states calculations was set as 0.5 *N*_e_ + *N*_i_, where *N*_e_ and *N*_i_ represent the number of valence electrons considered in the pseudopotential and atoms of the substrate. To further validate the d-band modulation mechanism, we performed additional density-of-states calculations for Pt_3_M surfaces using SCAN + rVV10. The rVV10 parameters were set to BPARAM = 15.7 and CPARAM = 0.0093, with other settings kept consistent with the optB88-vdW calculations. A clear linear correlation is maintained between the valence-electron-derived descriptor and the corresponding d-band center calculated with SCAN + rVV10 (Fig. S7).

Construction of adsorption models and post-processing analysis were carried out using an in-house, open-source Python infrastructure—the Dispersion-Inclusive Surface Chemistry Optimizer (DISCO) platform—designed for high-throughput simulations of surface and interfacial interactions in catalysis and advanced materials research. In particular, a periodic four-layer (111) slab was used to construct the substrate, with the bottom two layers fixed. Further increasing the surface thickness to six layers only creates energetic variations below 50 meV for the precursor states; for example, the adsorption energy of precursor benzene on Pt_3_Zr(111) is −1.14 eV for the four-layer slab and −1.10 eV for the six-layer slab. A vacuum layer exceeding 10 Å was included to avoid periodic interactions. To ensure the robustness of our classification, we systematically investigated multiple high-symmetry adsorption sites, including bridge30, fcc30, hcp30, top30, bridge0, fcc0, hcp0, and top0, for benzene on Pt_3_M(111) surfaces (Fig. S1). Based on this sampling, the energetically favorable bridge30 configuration was consistently used as the initial geometry for all adsorption systems reported in this work.

The climbing-image nudged elastic band (CI-NEB) method^67^ was used to calculate the diffusion and transition barriers of benzene on those alloy surfaces where five images were inserted to explore the transition states. Crystal orbital Hamilton population (COHP) and integrated COHP (ICOHP) analyses were performed using the LOBSTER package^68,69^ to obtain the interfacial bonding and anti-bonding states. To study the dynamic behavior of benzene molecules on these surfaces, *ab initio* molecular dynamics (AIMD) calculations were performed, utilizing a canonical NVT ensemble with temperatures set at 300 K. Five independent 10-ps AIMD simulations for benzene on a Pt_3_Zr system were performed with a step size of 2 fs.

The effective potential and transport calculations were carried out by the non-equilibrium Green's function (NEGF) method in the framework of DFT, implemented in Atomistix ToolKit (ATK) software, version 2018.06.^70^ The exchange-correlation functional was treated by the PBE formulation, together with FHI pseudopotentials with a double-ζ-polarized basis set (DZP) for other elements. A gold adatom surface was used as the tip to construct the transport model (Fig. S21). Since the tunneling current is exponentially related to the tip-molecule distance, a careful balance is required in the choice of electrode separation. In our transport property analysis, an electrode separation of 7.0 Å was employed, which represents an optimal compromise, avoiding configuration disturbance while ensuring a detectable current at low, safe biases (Fig. S22). Based on this configuration, we selected a modest operating bias window of ±0.1 V. The bias is sufficient to probe the transport characteristics of the junction while remaining safely below the threshold electric field for molecular reorientation.^3^ The Brillouin zone was sampled using a 3 × 3 × 135 Monkhorst–Pack grid for the two electrodes.

## Author contributions

S. Y.: conceptualization, data curation, formal analysis, funding acquisition, methodology, writing – original draft, writing – review & editing; J. Z.: data curation, writing – review & editing; Y. Z.: writing – review & editing; G. C.: writing – review & editing; J. R.: funding acquisition, writing – review & editing; W. L.: formal analysis, funding acquisition, writing – review & editing.

## Conflicts of interest

There are no conflicts to declare.

## Supplementary Material

SC-017-D5SC08297H-s001

## Data Availability

The data supporting this article have been included as part of the supplementary information (SI). Supplementary information: adsorption structures and energies; COHP analysis; PDOS plots; adsorption regime classification; linear fitting for *ε*_d_ data; diffusion pathway; transport model; intrinsic properties of elements. See DOI: https://doi.org/10.1039/d5sc08297h.
