# Supplementary material for: A predictive descriptor for the d-band center in intermetallic alloys accelerates the design of robust molecular switches
Source: Chem Sci. 2026 Jan 13;17(10):5229–39. doi: 10.1039/d5sc08297h (PMC12817911; doi:10.1039/d5sc08297h)
Supplement: SC-017-D5SC08297H-s001 [file SC-017-D5SC08297H-s001.pdf]

Supplementary Information (SI) for Chemical Science.
This journal is © The Royal Society of Chemistry 2026

Supplementary Information for

A Predictive Descriptor for d-Band Center in Intermetallic Alloys Accelerates the Design of Robust Molecular Switches

Sha Yang[1]*, *Junjun Zhou*[1], *Yirong Zhang*[1], *Guolin Cao*[1], *Ji-Chang Ren*[1], *Wei Liu*[2]*

[1]Nano and Heterogeneous Materials Center, School of Materials Science and Engineering, Nanjing University of Science and Technology, Nanjing 210094, Jiangsu, China.

[2]State Key Laboratory of Rare Earth Resource Utilization, Changchun Institute of Applied Chemistry, Chinese Academy of Sciences, Changchun 130022, China.

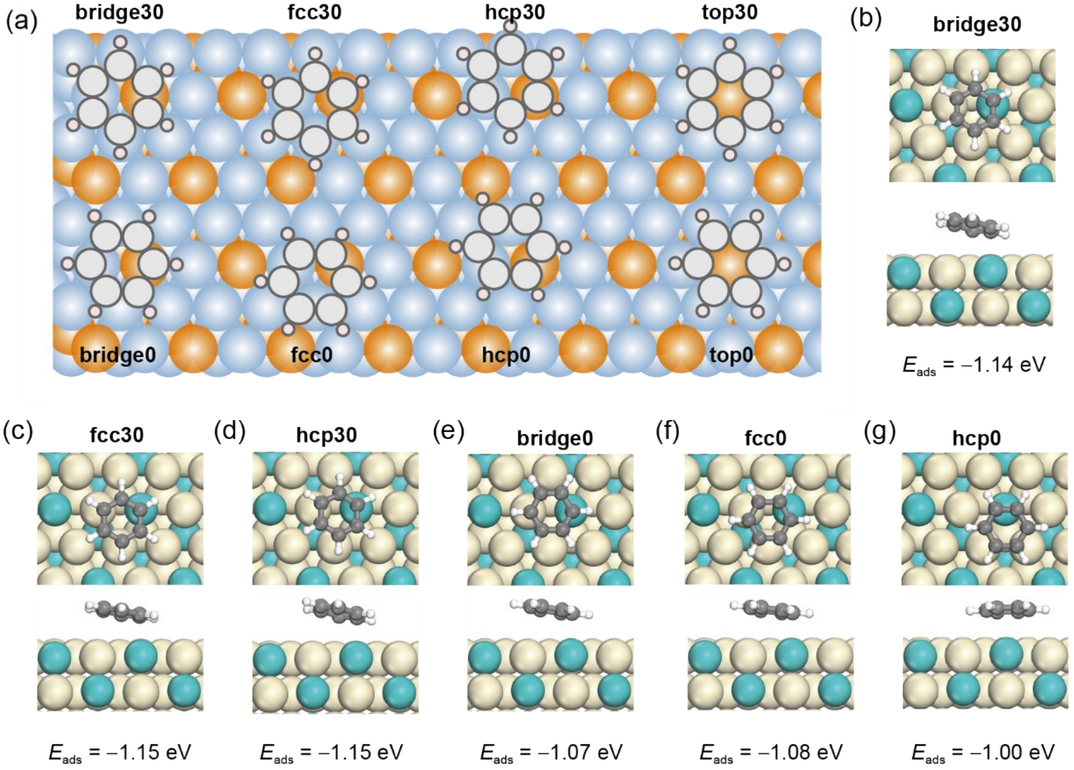

Figure S1. (a) Initial adsorption sites considered for benzene on the alloyed surfaces. The blue and yellow atoms denote the host and guest metals in A_3B. "30" and "0" denote the rotation angle of the C-C bond relative to the underlying metal-metal bond. (b)-(g) Optimized structures and corresponding adsorption energies of benzene in the precursor state at different high-symmetry sites on $Pt_3Zr(111)$.

Using Pt_3Zr as a representative system, we observed that benzene relaxes into a type-II adsorption regime at six of these sites (bridge30, fcc30, hcp30, bridge0, fcc0, and hcp0). Although a purely chemisorbed state is observed at the top0 and top30 sites, their adsorption energies are at least 0.2 eV higher than those of the type-II regime, in agreement with the preferred adsorption sites of benzene on pure Pt surfaces[1]. Among the mixed-bonding precursor states, the bridge30, fcc30, and hcp30 configurations exhibit similar stability and are energetically more favorable than their corresponding "0" counterparts. Given the structural similarity of bridge30 to both fcc30 and hcp30, we have selected the bridge30 configuration as the reference geometry for all adsorption systems reported in this study. This systematic sampling confirms that the classification into type-I and type-II regimes is not an artifact of site selection but rather reflect the intrinsic interaction between the benzene molecule and the alloy surface.

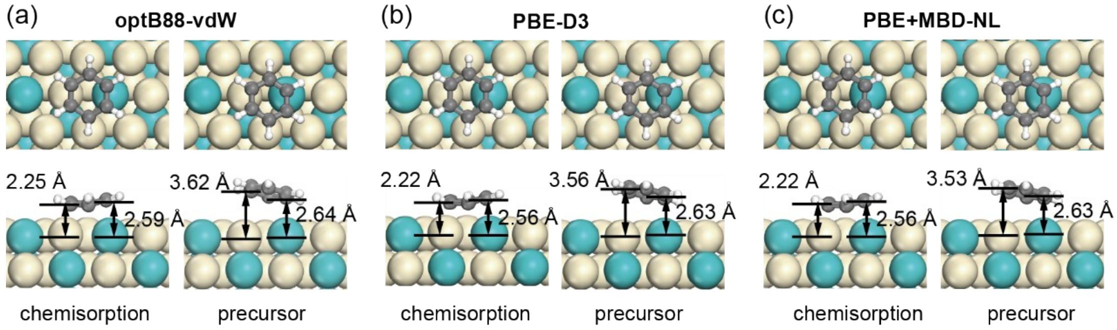

Figure S2. Comparison of relaxed geometries for benzene on $Pt_3Zr(111)$ calculated by optB88-vdW, PBE-D3, and PBE+MBD-NL methods. The values represent the C-Pt (left) and C-M (right) distances.

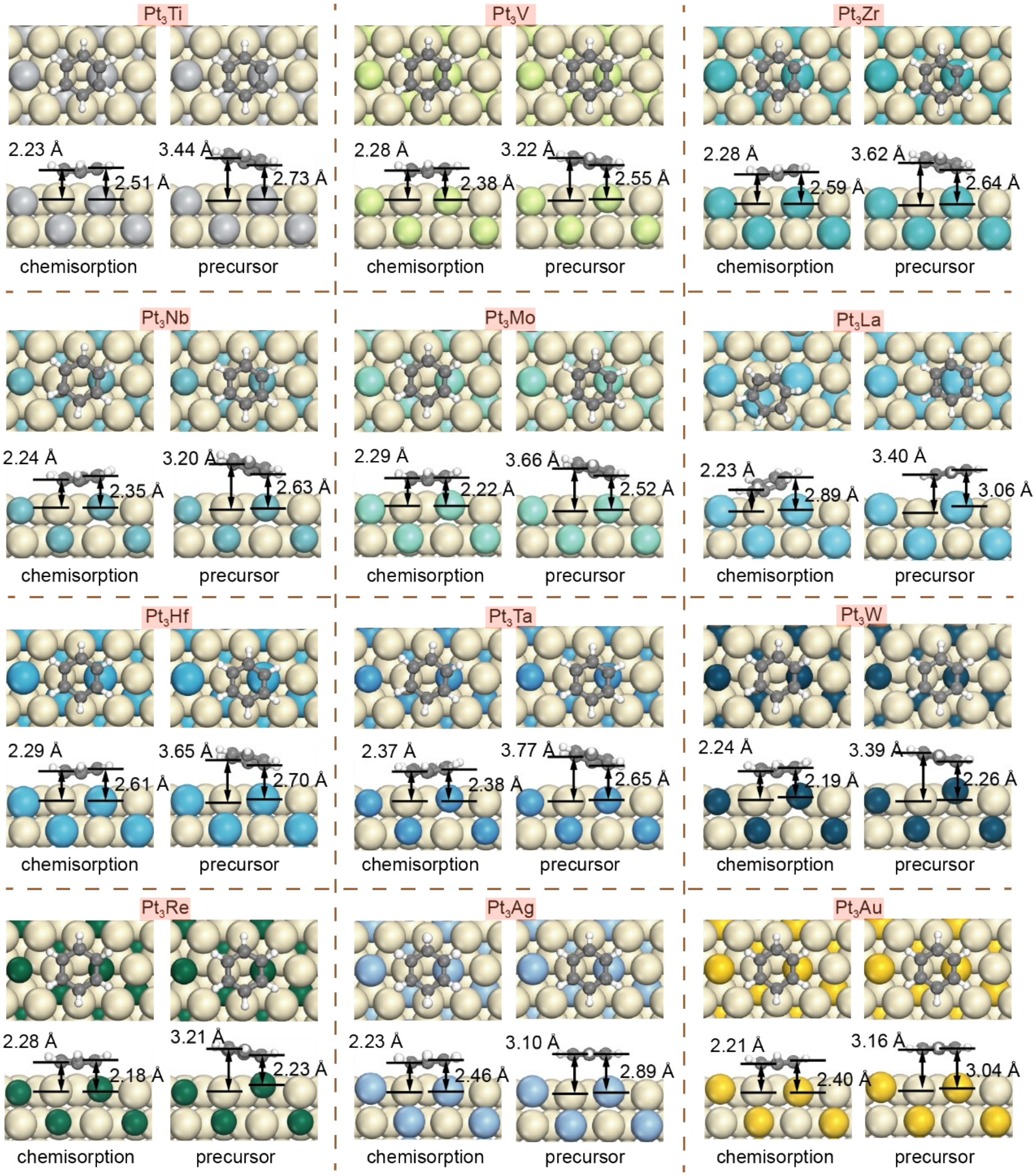

Figure S3. OptB88-vdW-calculated bistable adsorption structures of benzene on Pt_3M surfaces (M = Ti, V, Zr, Nb, Mo, La, Hf, Ta, W, Re, Ag, Au). The labeled distances indicate the separations between the carbon atoms and the underlying substrate atoms. The C-Pt distance for benzene on Pt(111) is 2.21 Å.

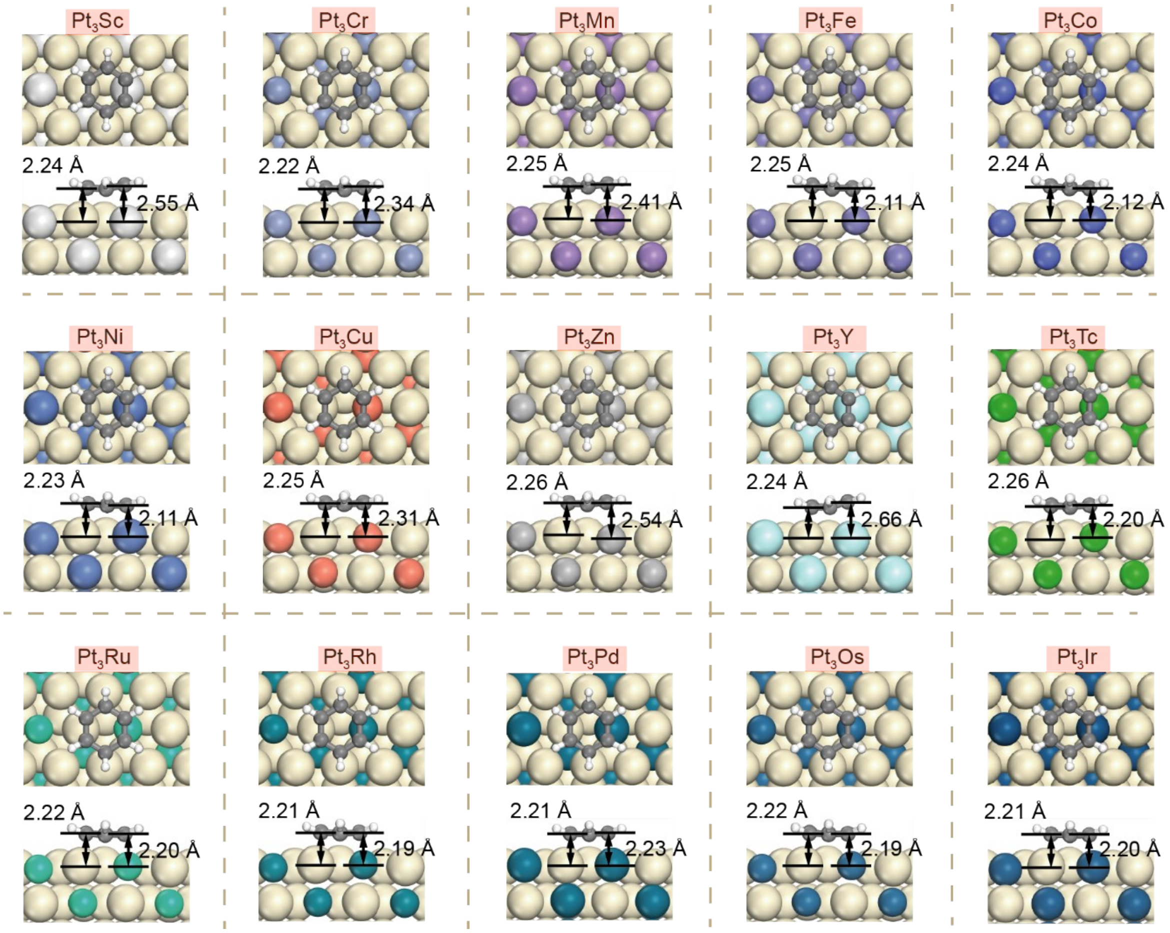

Figure S4. OptB88-vdW-calculated monostable chemisorption structures of benzene on Pt_3M surfaces (M = Sc, Cr, Mn, Fe, Co, Ni, Cu, Zn, Y, Tc, Ru, Rh, Pd, Os, Ir). The labeled distances correspond to the separations between the carbon atoms and the underlying metal substrate.

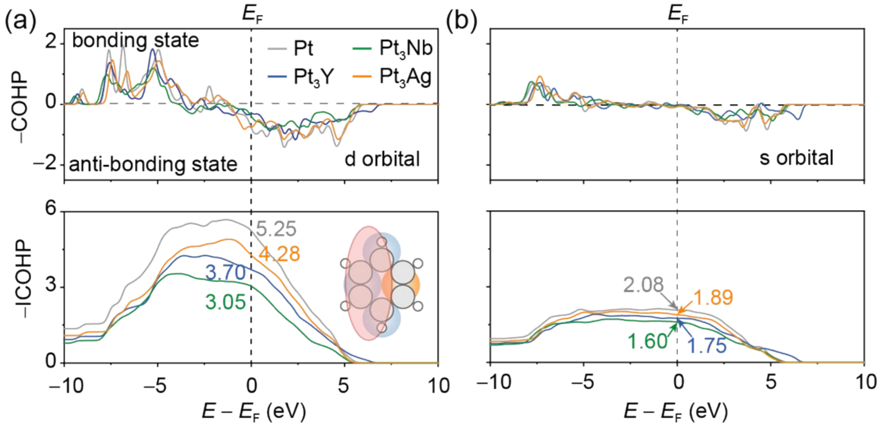

Figure S5. Crystal orbital Hamilton population (COHP) and integrated COHP (ICOHP) between C atoms and d- (a) or s-orbitals (b) of below Pt atoms for benzene on the (111) surfaces of Pt (gray), Pt_3Y (blue), Pt_3Nb (green), and Pt_3Ag (orange). The inset shows the C-Pt pairs considered in calculations. The −ICOHP values in the lower panel were obtained by integrating the Hamiltonian-weighted band energy from $-\infty$ to 0. The energy scale is referenced to the Fermi level ($E_F = 0$).

The modulation of the Pt d-band significantly influences the molecule-Pt bonding strength. As the d-band center shifts downward, the value of −ICOHP at the Fermi level decreases, indicating a weakening of covalent bonding between carbon atoms and Pt. This electronic structure change correlates with the observation that the potential energy landscape above Pt sites exhibits two local energy minima. We also evaluated the contribution of s-orbitals to the interfacial bonding but found only minor differences across the adsorption systems. Furthermore, although Pt(111) exhibits a higher d-band center than Pt_3Y and Pt_3Ag, the C-Pt bonding strength is stronger in Pt(111). This can be attributed to the larger covalent radii of Y and Ag, which increase the C-Pt distance and thereby weaken the bonding interaction.

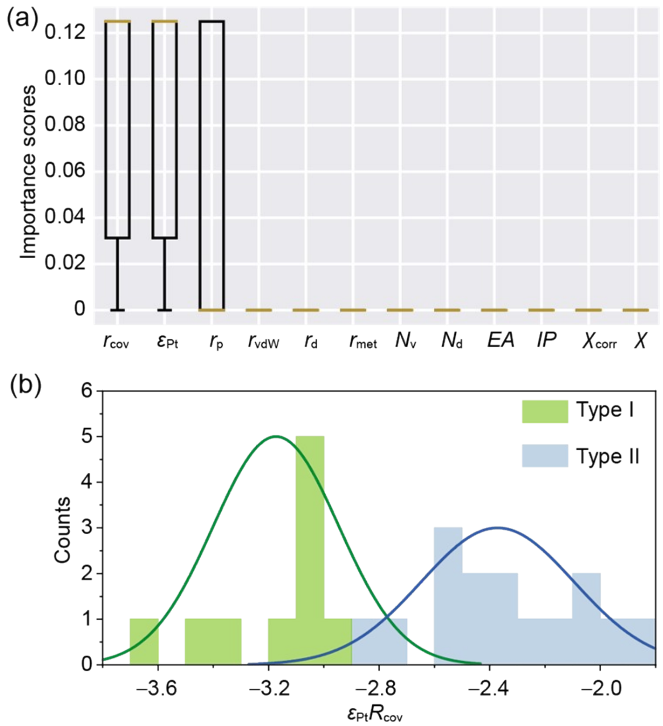

Figure S6. (a) Feature importance scores of descriptors for predicting adsorption regimes of benzene on $Pt_3M(111)$ surfaces using the random forest classifier. (b) Classification model of adsorption regimes based on a descriptor of $\varepsilon_{Pt}R_{cov}$, where ε_{Pt} and R_{cov} represent the d-band center of surface Pt atoms and normalized covalent radius ($r_{cov(M)}/r_{cov(Pt)}$).

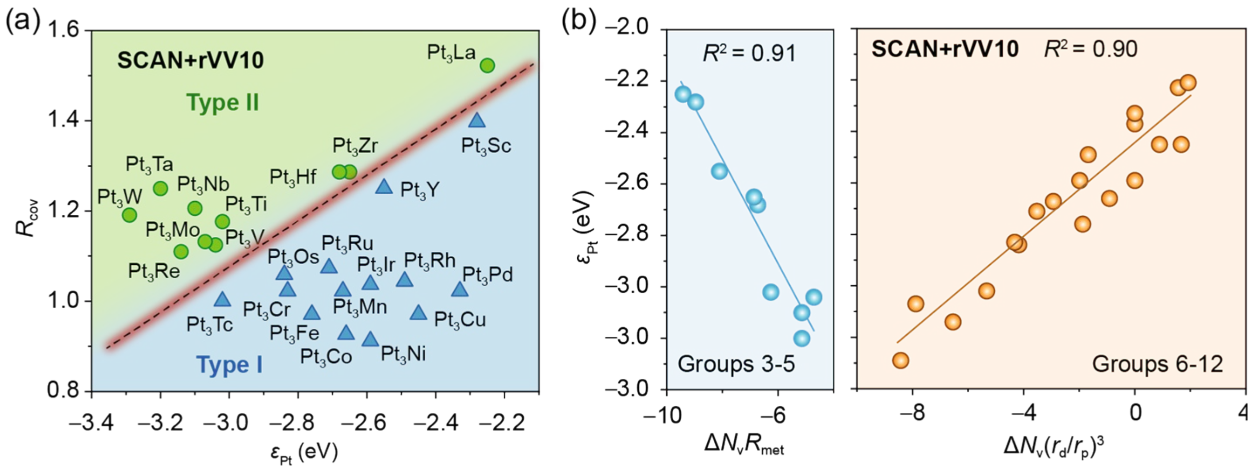

Figure S7. (a) Classification of benzene adsorption regimes on Pt_3M surfaces based on the normalized covalent radius (R_{cov}) and Pt d-band center (ε_{Pt}) obtained from SCAN+rVV10 calculations. (b) Linear correlation between the valence electron-derived descriptors and the DFT-calculated ε_{Pt} for Pt_3M systems, using SCAN+rVV10 methods.

The SCAN+rVV10 method has been shown to accurately predict benzene adsorption energies on coinage metal surfaces[2], though it requires more than twice the computational cost of optB88-vdW. As shown in Figure S7a, the classification of adsorption regimes (type-I vs. type-II) based on the SCAN+rVV10-calculated Pt d-band center (ε_{Pt}) plotted against the normalized covalent radius, $R_{cov} = r_{cov}(M)/r_{cov}(Pt)$, remains fully consistent with the results obtained using optB88-vdW (detailed relaxed structures are provided in Figures S8 and S9). Systems in the top-left region correspond to type-II adsorption, while those in the bottom-right region belong to type I. Furthermore, the descriptor derived from optB88-vdW calculations well predicts the ε_{Pt} values obtained with SCAN+rVV10 (Figure S7b). These results confirm that our central findings—the adsorption regime classification and the predictive power of the electronic descriptor—are robust with respect to the choice of advanced functionals.

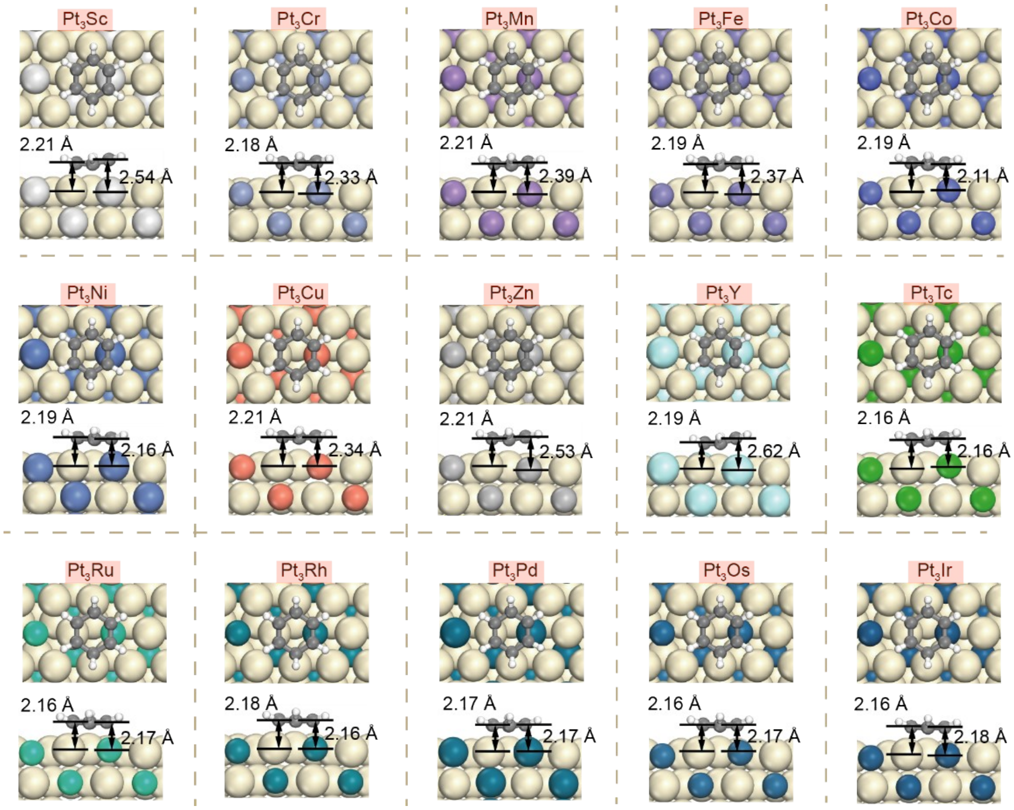

Figure S8. SCAN+rVV10-calculated monostable chemisorption structures of benzene on Pt_3M surfaces (M = Sc, Cr, Mn, Fe, Co, Ni, Cu, Zn, Y, Tc, Ru, Rh, Pd, Os, Ir). The labeled distances correspond to the separations between the carbon atoms and the underlying metal substrate.

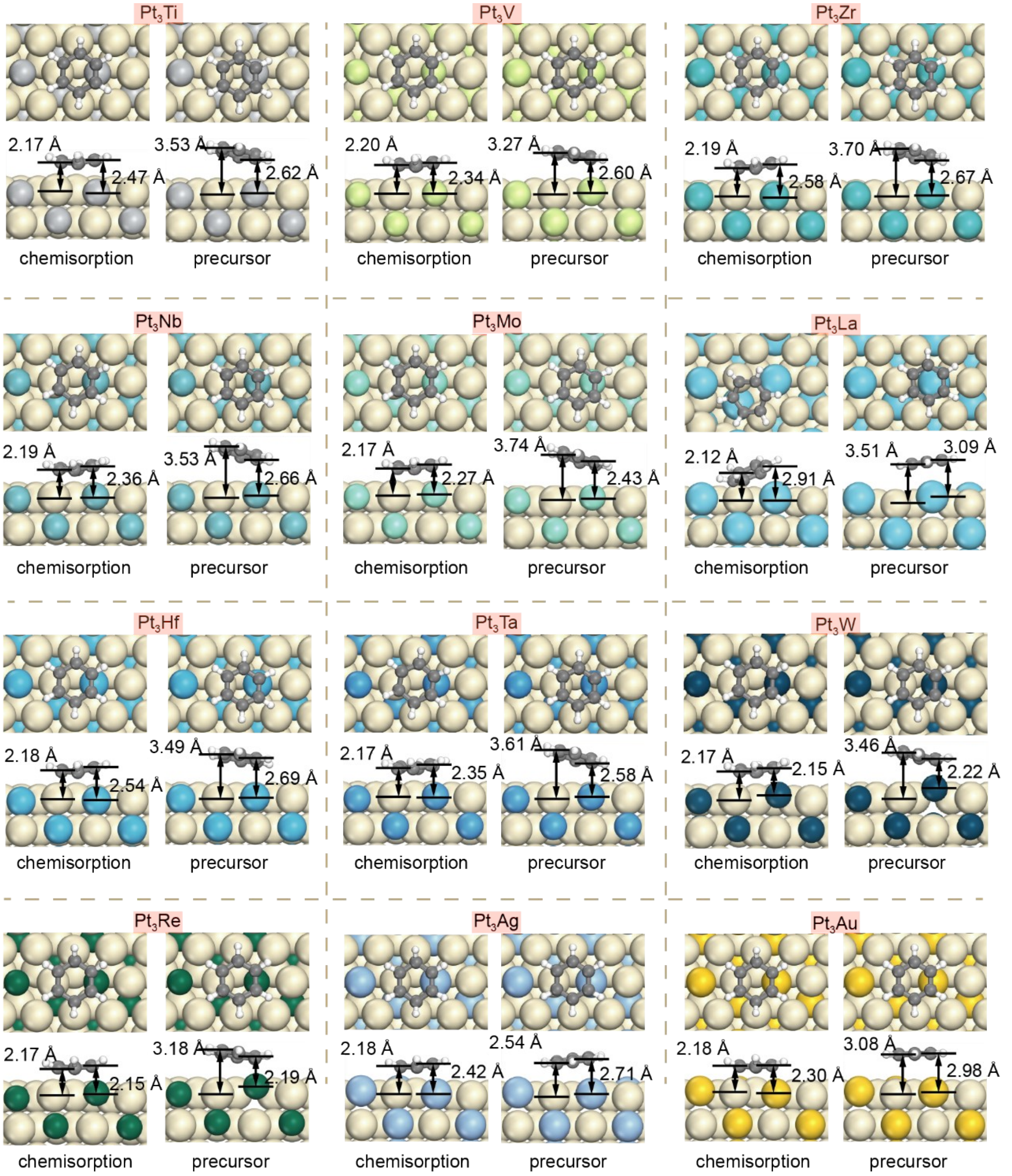

Figure S9. SCAN+rVV10-calculated bistable adsorption structures of benzene on Pt_3M surfaces (M = Ti, V, Zr, Nb, Mo, La, Hf, Ta, W, Re, Ag, Au). The labeled distances indicate the separations between the carbon atoms and the underlying substrate atoms.

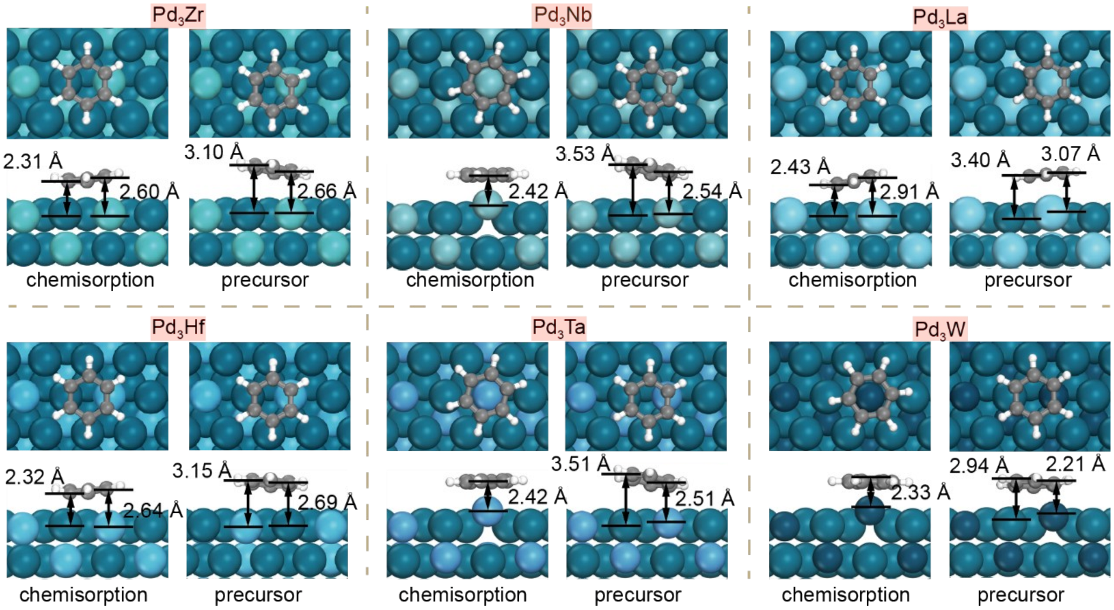

Figure S10. Bistable adsorption structures of benzene on $Pd_3M(111)$ surfaces. The values represent the distances between the carbon atoms and the below metal atoms.

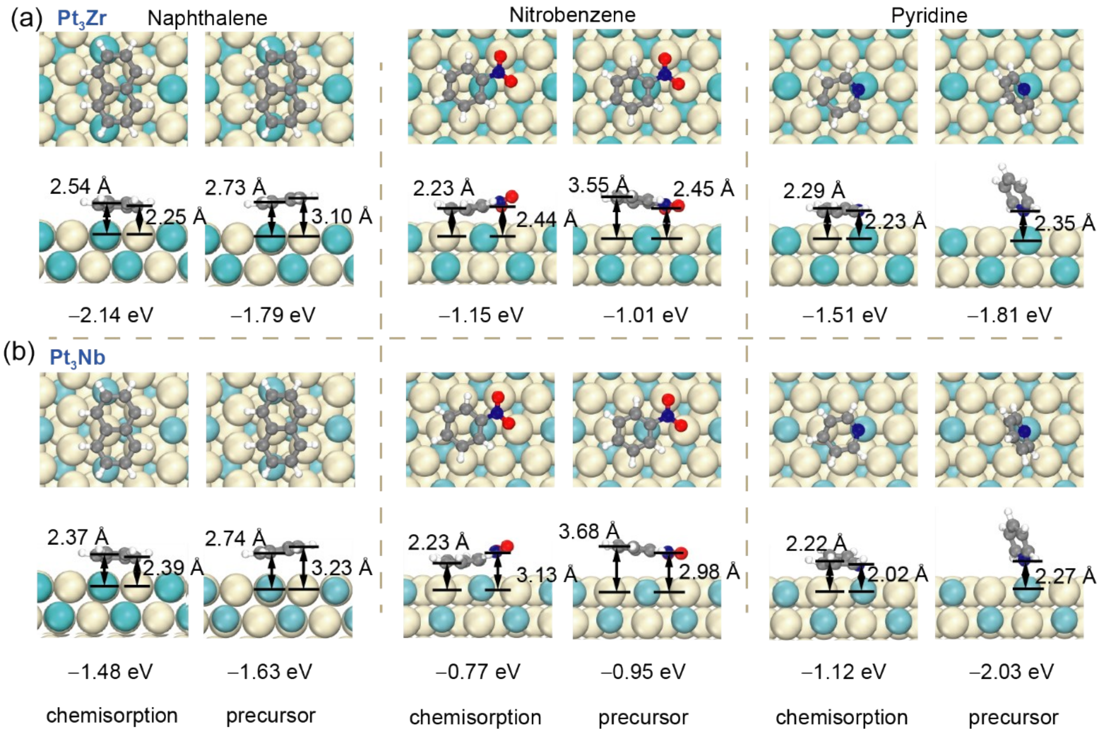

Figure S11. Adsorption structures and corresponding energies of naphthalene, nitrobenzene, and pyridine on the (111) surfaces of Pt_3Zr (a) and Pt_3Nb (b) alloys. The labeled distances represent the separation between the molecules and the underlying metal atoms.

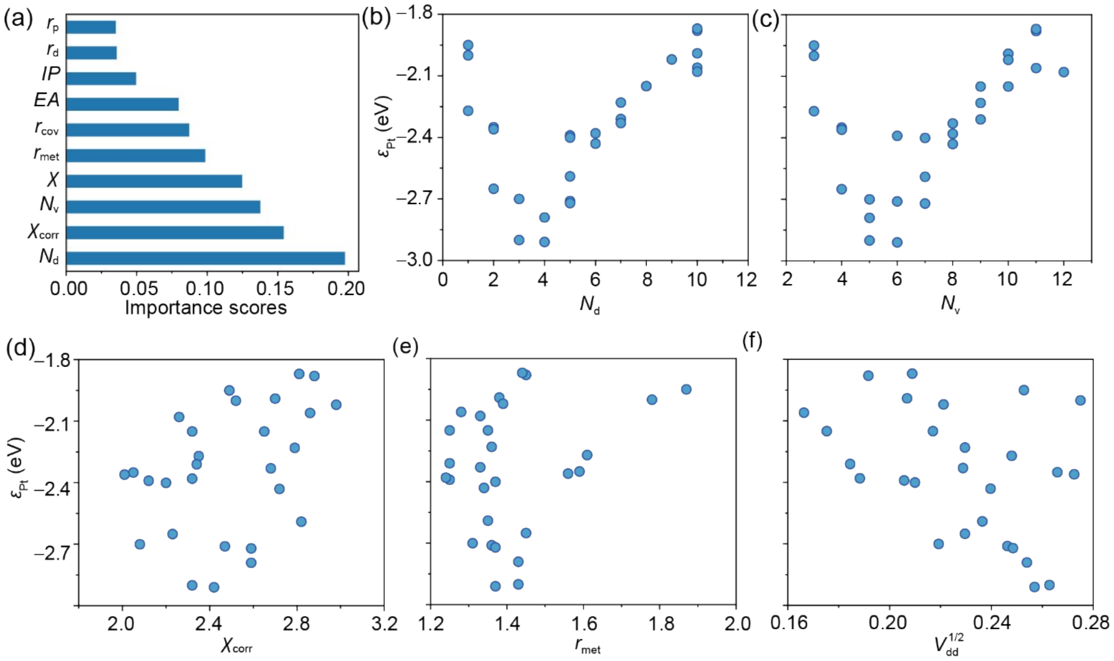

Figure S12. (a) Feature importance scores of descriptors for prediction of d-band center using the random forest regression. (b)-(e) Correlation between Pt d-band centers and various parameters, including free-atom d-band filling (N_d), valence electron number (N_v), corrected electronegativity (χ_{corr}), metallic radius (r_{met}) of guest metals. These parameters can be seen in Table S2. (f) Correlation between Pt d-band center and matrix elements between d orbitals.

When metal A interact with metal B, the matrix elements between the d orbitals can be estimated by[3] $V_{ddm} = \eta_{ddm}\hbar^2(r_{d(A)}r_{d(B)})^{3/2}/(m_e d^5)$, where η_{ddm} can be seen as a constant, $r_{d(A)}$ and $r_{d(B)}$ denote the d state radii of metals A and B, the m_e is the electron mass, and d is the internuclear distance (Table S4). The value of $\hbar^2/m_e$ is 7.62 eV·Å^2. For simplicity, the constant η_{ddm} was omitted and the square root of $\hbar^2(r_{d(A)}r_{d(B)})^{3/2}/(m_e d^5)$ was used[4].

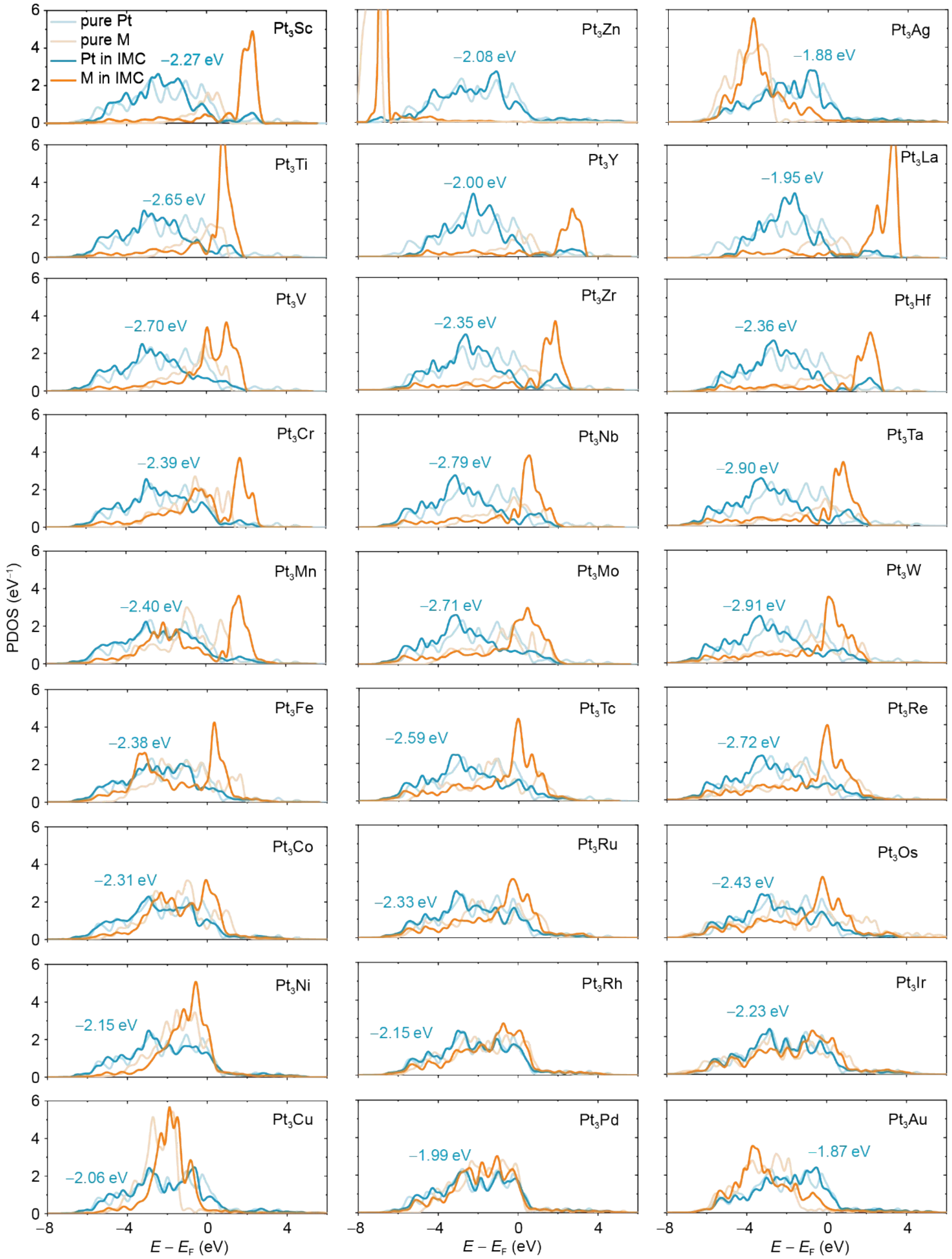

Figure S13. Projected density of states (PDOS) of Pt and guest metal atoms in (111) surface of Pt_3M, represented by the blue and orange lines. The light-colored lines denote the PDOS of the pure metal. The inserted values represent the d band center of surface Pt atoms. The energy scale is referenced to the Fermi level ($E_F = 0$).

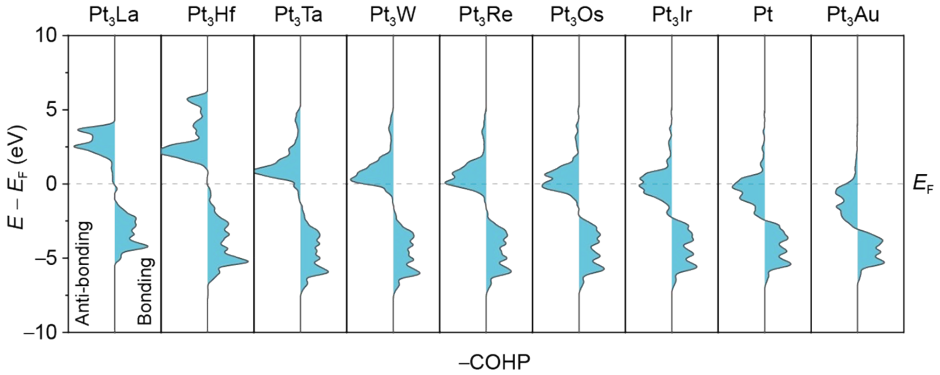

Figure S14. COHP plots for Pt-M 5d-5d orbital interactions, where M denote the 5d metals. Similar to Pt_3M with 3d/4d guest metals, the d-d anti-bonding states begin to occupy below the Fermi level at group 5 (Pt_3Ta). The energy scale is referenced to the Fermi level ($E_F = 0$).

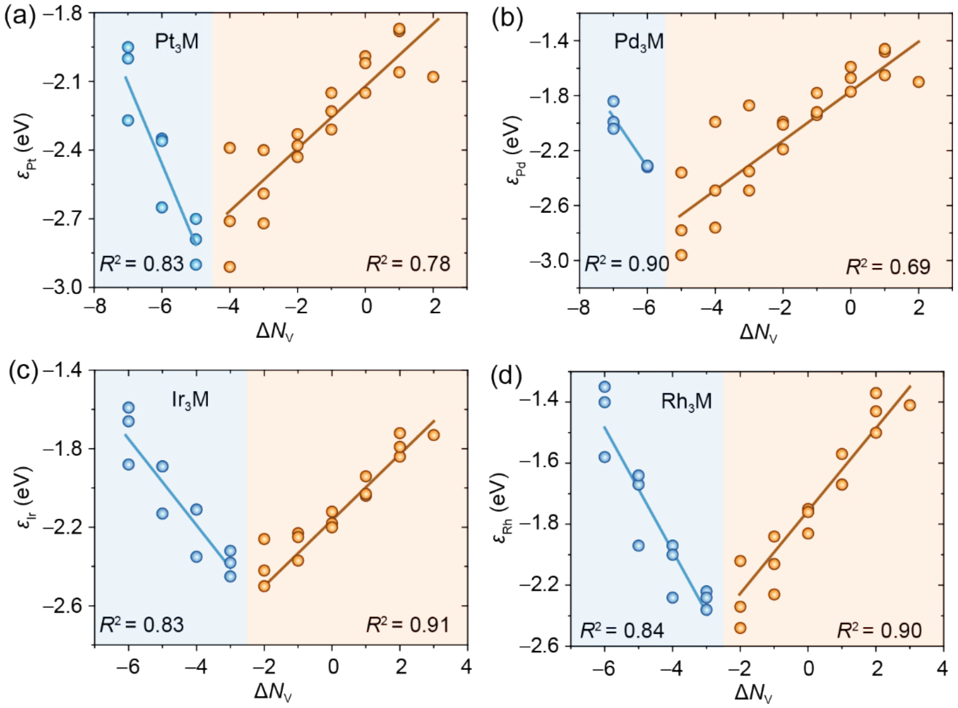

Figure S15. Correlation between d-band center of the host metal and the valence electron number of the guest metal in Pt_3M, Pd_3M, Ir_3M, and Rh_3M.

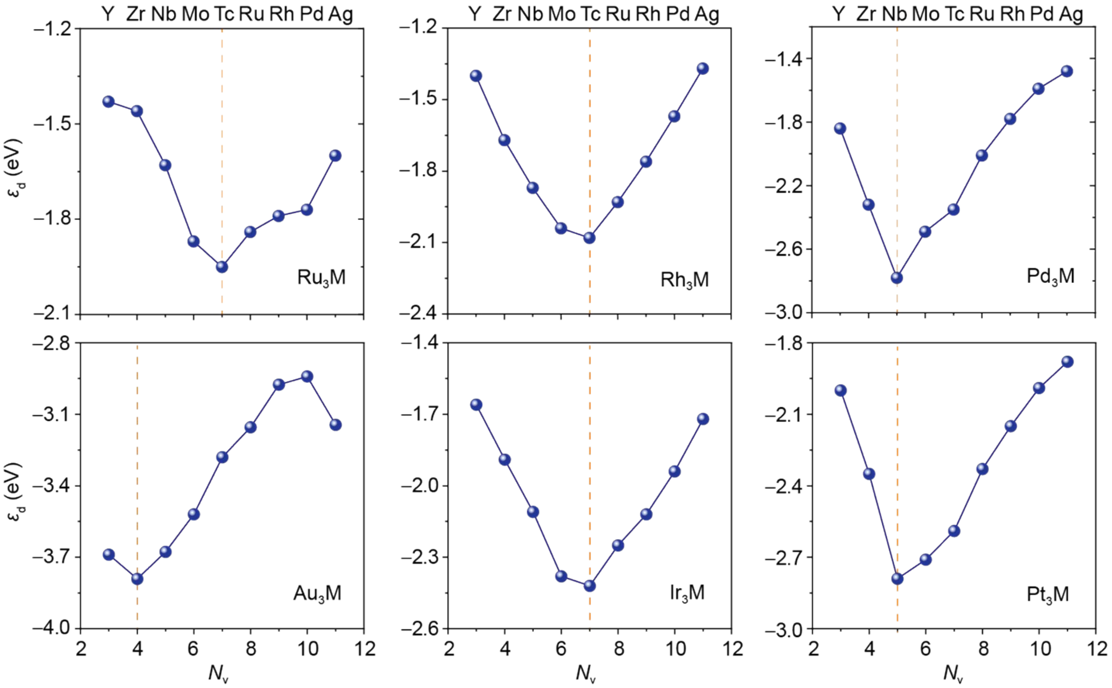

Figure S16. Host metal's d-band center as a function of the valence electron number of the guest metal for various A_3B alloys. The orange dashed line denote the position of the minima d-band center.

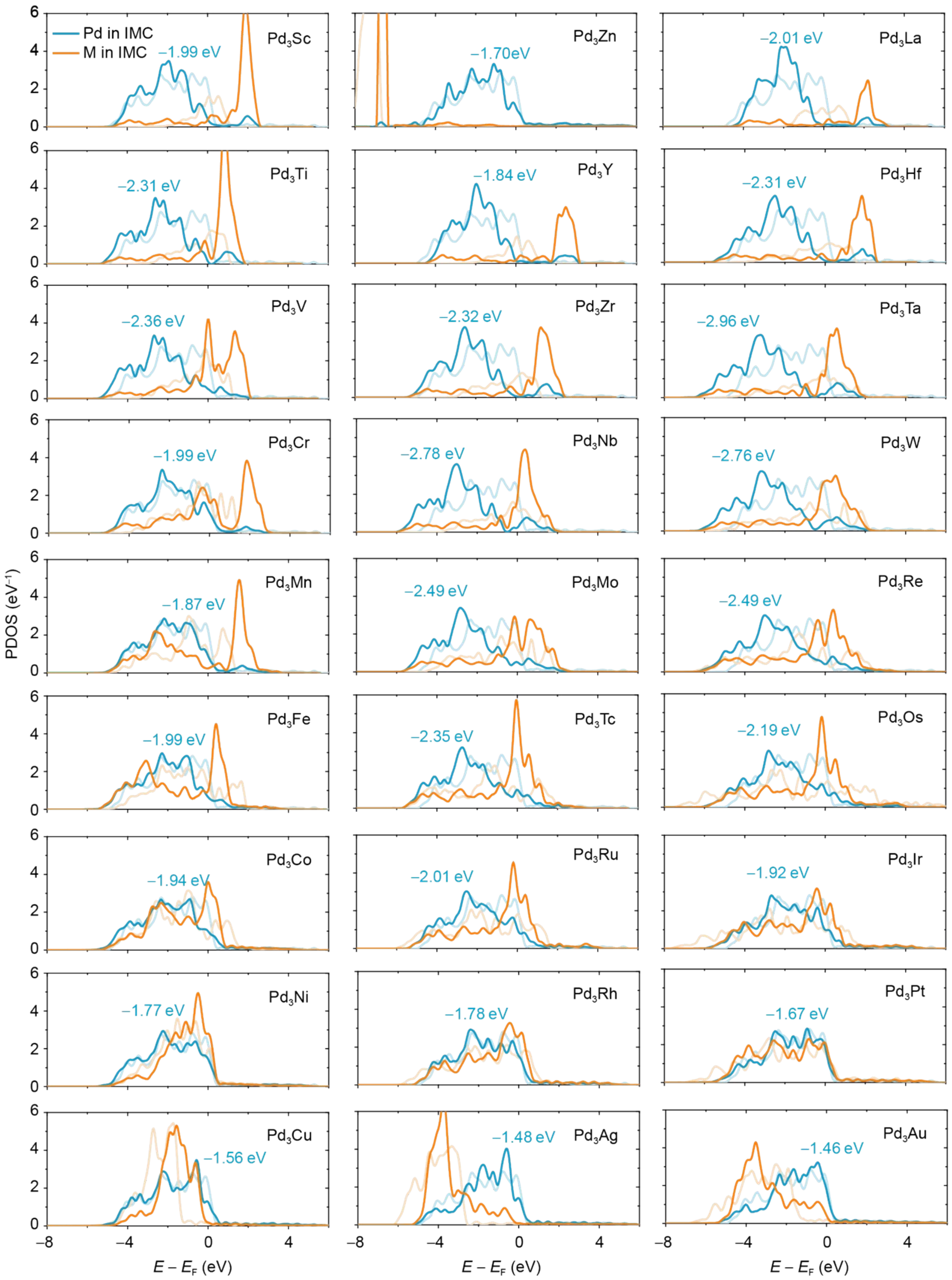

Figure S17. PDOS of Pd and guest metals on (111) surfaces of Pd_3M, represented by the blue and orange lines. The light-colored lines denote the PDOS of their pure counterparts. The inserted values represent the d band center of surface Pd atoms. The energy scale is referenced to the Fermi level ($E_F = 0$).

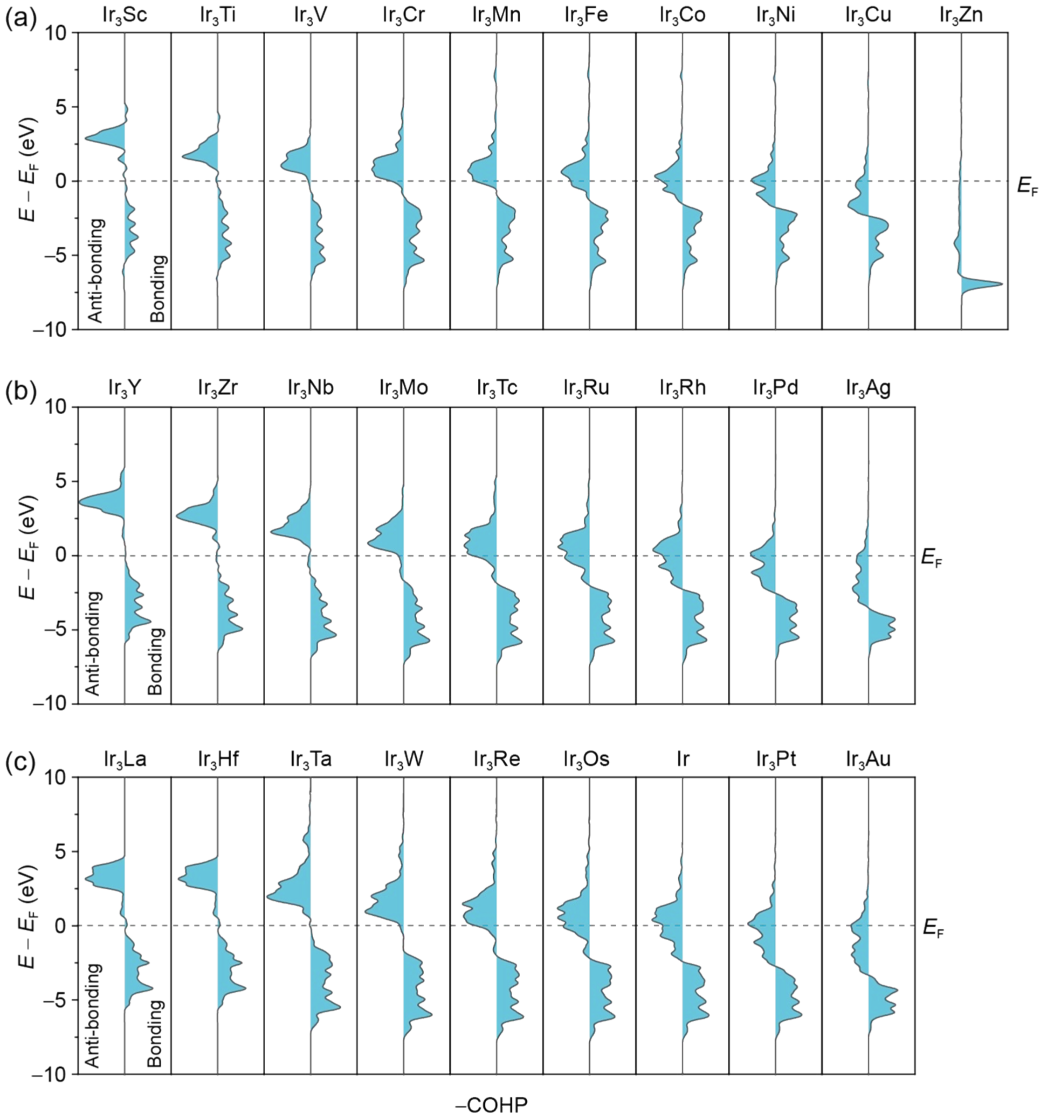

Figure S18. COHP for Pd-M d-d orbital interactions, where M denote the 3d, 4d, and 5d metals. The energy scale is referenced to the Fermi level ($E_F = 0$).

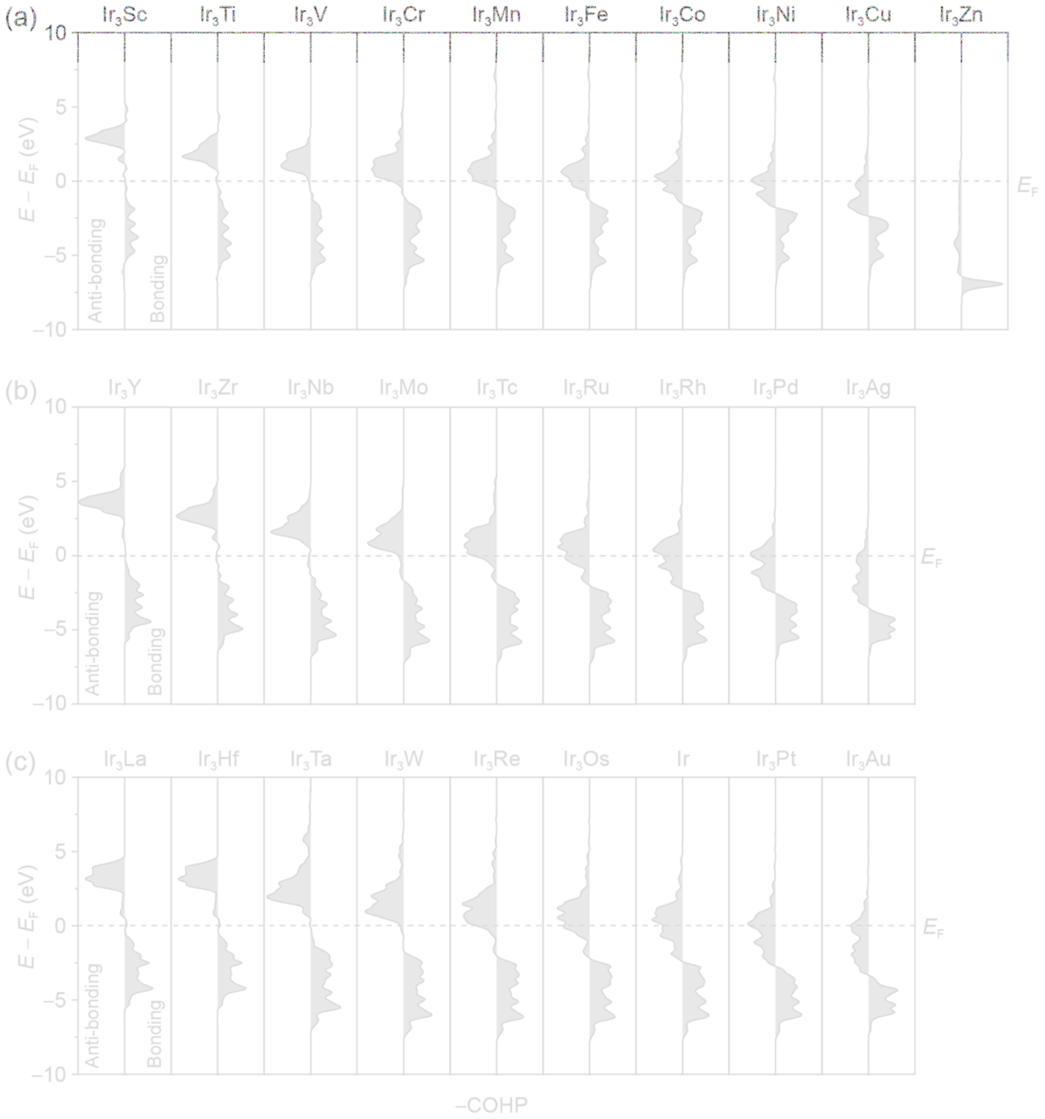

Figure S19. COHP for Ir-M d-d orbital interactions, where M denote the 3d, 4d, and 5d metals. The energy scale is referenced to the Fermi level ($E_F = 0$).

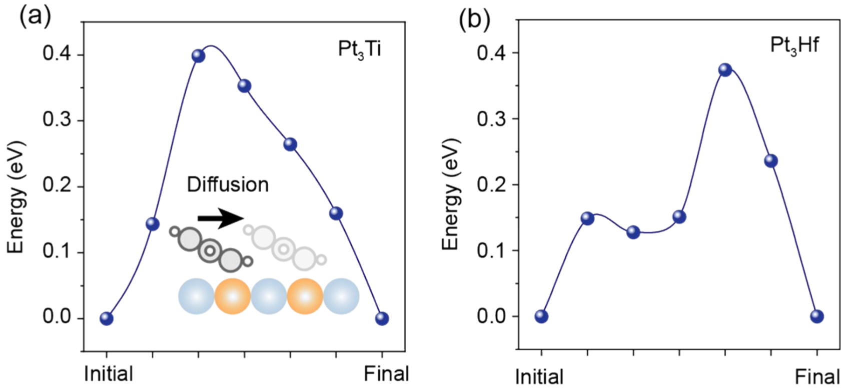

Figure S20. Diffusion barrier of precursor benzene on (111) surfaces of Pt_3Ti and Pt_3Hf alloys by CI-NEB method. The inset illustrates the diffusion pathway between adjacent precursor sites.

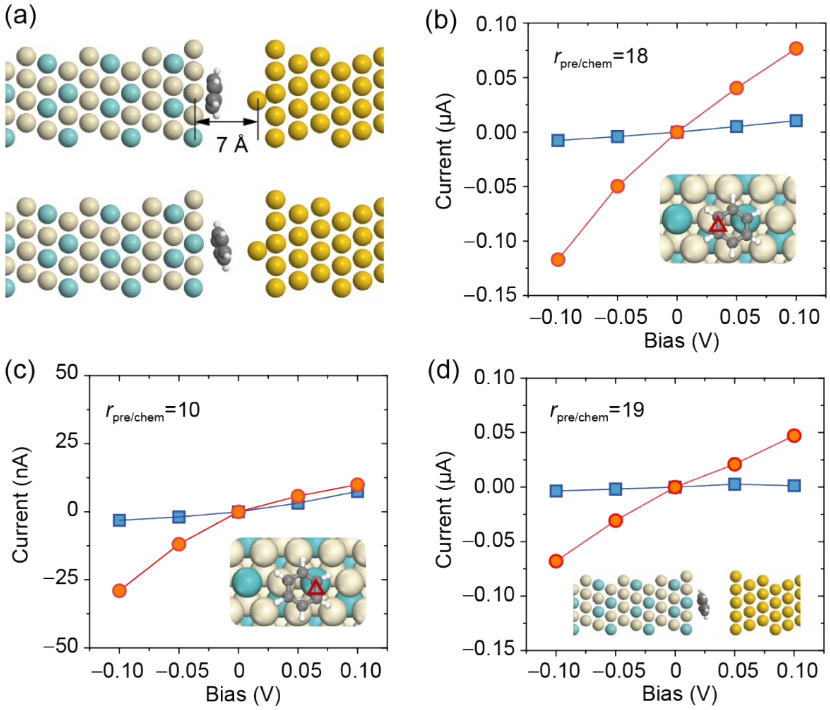

Figure S21. (a) Transport model in current calculations for bistable adsorption regime of benzene/Pt_3Zr, where the tip was simulated by a gold adatom on Au(111). The distance between the Pt3Zr surface and the gold tip is set to be 7 Å to avoid the disturbance of the tip to the adsorption structure. (b)-(c) Current versus bias with the tip on top of different position, denoted by the red triangle in the inset. (d) Current versus bias with a planar gold electrode, as illustrated in the inset.

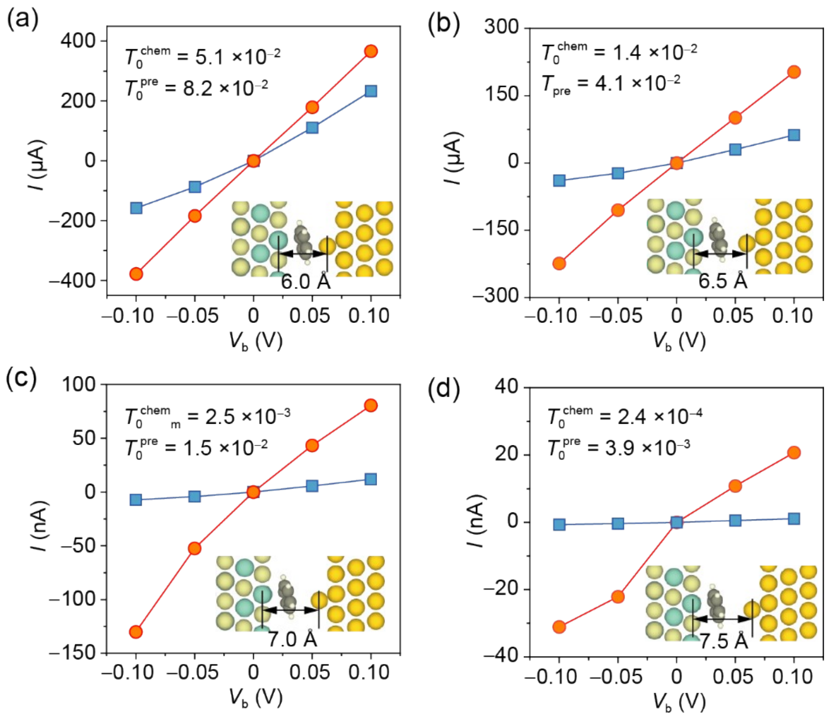

Figure S22. Current-voltage characteristics Pt_3Zr/benzene/Au junctions with tip-substrate separations of 6.0 Å (a), 6.5 Å (b), 7.0 Å (c), and 7.5 Å (d). The zero-bias transmission values at the Fermi level are provided in each panel.

In our study, the transport models were constructed to simulate scanning tunneling microscopy (STM) measurements, where the current is dominated by electron tunneling between a metallic tip and the molecule-substrate complex[5]. For this geometry, the tunneling current follows $I \propto \underline{V}_b \exp(-A\varphi^{1/2}s)$, where V_b is the bias voltage, φ is the tunneling barrier, s is the tip-molecule distance, and A is a constant[6]. This exponential dependence requires a careful balance in the choice of electrode separation: overly small separations can perturb the adsorption configuration, whereas excessively large separation leads to currents that are impractically small within a safe bias window.

To determine an optimal electrode separation, we performed systematic non-equilibrium Green's function (NEGF) calculations for tip-substrate distances ranging from 6.0 to 7.5 Å (Figure R2). At short separations ($\leqslant$ 6.5 Å), the resulting tip-molecule distance is below 3.5 Å, leading to significant electronic perturbation and an ohmic-like response for the precursor state (orange data points in Figure S22a and S22b). At a separation of 7.0 Å, the tip-molecule distance exceeds 4.0 Å, effectively minimizing tip-induced disturbance while maintaining a measurable tunneling current. Further increasing the separation to 7.5 Å strongly suppresses electron tunneling in both

the chemisorbed and precursor states, as evidenced by the substantially reduced zero-bias transmission values at the Fermi level (T_0). Such large separations would require higher operating biases to achieve detectable currents, thereby increasing the risk of field-induced molecular rotation. Accordingly, an electrode separation of 7.0 Å represents an optimal compromise between minimizing structural perturbation and ensuring reliable current detection. Based on this configuration, we selected a modest operating bias window of ±0.1 V. The bias is sufficient to probe the transport characteristics of the junction while remaining safely below the threshold electric field for molecular reorientation (compare, e.g., the ~0.7 V threshold reported in $Sc_2C_2@C_{88}$ switches[7]).

Table S1. Adsorption energies and the corresponding vdW contributions for benzene on the (111) surfaces of Pt_3Ti, Pt_3V, Pt_3Zr, Pt_3Nb, Pt_3Mo, Pt_3La, Pt_3Hf, Pt_3Ta, Pt_3W, Pt_3Re, Pt_3Ag, and Pt_3Au.

Alloy	Chemisorption		Precursor	
	E_{ads} (eV)	E_{vdW} (eV)	E_{ads} (eV)	E_{vdW} (eV)
Pt_3Ti	−1.18	−2.54	−1.00	−1.55
Pt_3V	−1.39	−2.64	−1.04	−1.74
Pt_3Zr	−1.19	−2.48	−1.14	−1.57
Pt_3Nb	−0.79	−2.54	−1.10	−1.50
Pt_3Mo	−1.24	−2.63	−1.10	−1.55
Pt_3La	−2.27	−2.34	−1.32	−1.57
Pt_3Hf	−1.18	−2.51	−1.12	−1.50
Pt_3Ta	−0.71	−2.61	−1.08	−1.55
Pt_3W	−1.17	−2.48	−1.21	−1.30
Pt_3Re	−1.64	−2.73	−1.20	−1.81
Pt_3Ag	−1.08	−2.62	−1.01	−1.62
Pt_3Au	−1.25	−2.70	−1.00	−1.52

Table S2. Pauli electronegativity (χ, in $eV^{1/2}$)[8], corrected electronegativity (χ_{corr})[9], ionic potential (IP, in eV)[8], electronic affinity (EA, in eV)[8], metallic radius (r_{met}, in Å)[10], covalent radius (r_{cov}, in Å)[11], van der Waals radius (r_{vdW}, in Å), orbital radii (r_p and r_d, in Å)[12], d orbital occupancy (N_d), and valence number (N_v) of the elements in groups 3-12.

Element	χ	χ_{corr}	IP	EA	r_{met}	r_{cov}	r_{vdW}	r_p	r_d	N_d	N_v
Sc	1.36	2.35	6.56	0.19	1.61	1.70	2.58	0.50	0.54	1	3
Ti	1.54	2.23	6.83	0.08	1.45	1.60	2.46	0.47	0.49	2	4
V	1.63	2.08	6.75	0.53	1.31	1.53	2.42	0.44	0.45	3	5
Cr	1.66	2.12	6.77	0.67	1.25	1.39	2.45	0.42	0.43	5	6
Mn	1.55	2.20	7.43	0.00	1.37	1.39	2.45	0.39	0.39	5	7
Fe	1.83	2.32	7.90	0.15	1.24	1.32	2.44	0.37	0.37	6	8
Co	1.88	2.34	7.88	0.66	1.25	1.26	2.40	0.36	0.34	7	9
Ni	1.91	2.32	7.64	1.16	1.25	1.24	2.40	0.34	0.33	8	10
Cu	1.90	2.86	7.73	1.24	1.28	1.32	2.38	0.33	0.31	10	11
Zn	1.65	2.26	9.39	0.00	1.33	1.22	2.39	0.31	0.29	10	12
Y	1.22	2.52	6.22	0.31	1.78	1.90	2.75	0.65	0.87	1	3
Zr	1.33	2.05	6.63	0.43	1.59	1.75	2.52	0.61	0.79	2	4
Nb	1.60	2.59	6.76	0.90	1.43	1.64	2.56	0.59	0.75	4	5
Mo	2.16	2.47	7.09	0.75	1.36	1.54	2.45	0.56	0.70	5	6
Tc	1.90	2.82	7.28	0.55	1.35	1.47	2.44	0.54	0.65	5	7
Ru	2.20	2.68	7.36	1.05	1.33	1.46	2.46	0.52	0.62	7	8
Rh	2.28	2.65	7.46	1.14	1.35	1.42	2.44	0.50	0.59	8	9
Pd	2.20	2.70	8.34	0.56	1.38	1.39	2.15	0.48	0.57	10	10
Ag	1.93	2.88	7.58	1.30	1.45	1.45	2.53	0.46	0.54	10	11
La	1.10	2.49	5.77	0.50	1.87	2.07	2.98	0.83	1.09	1	3
Hf	1.30	2.01	6.83	0.00	1.56	1.75	2.53	0.62	0.83	2	4
Ta	1.50	2.32	7.89	0.32	1.43	1.7	2.57	0.60	0.78	3	5
W	2.36	2.42	7.98	0.86	1.37	1.62	2.49	0.58	0.75	4	6
Re	1.90	2.59	7.88	0.15	1.37	1.51	2.48	0.57	0.73	5	7
Os	2.20	2.72	8.70	1.10	1.34	1.44	2.41	0.55	0.70	6	8
Ir	2.20	2.79	9.10	1.57	1.36	1.41	2.29	0.54	0.68	7	9
Pt	2.28	2.98	9.00	2.13	1.39	1.36	2.32	0.52	0.66	9	10
Au	2.54	2.81	9.23	2.31	1.44	1.36	2.45	0.51	0.63	10	11

Table S3. Fillings (n_d) of d-band of surface Pt atoms in Pt_3M alloy with various guest M metal. The d-band filling of pure Pt is 8.02 electrons.

Element	n_d	Element	n_d	Element	n_d
Sc	8.096	Zn	8.093	Ag	8.058
Ti	8.054	Y	8.132	La	8.161
V	8.047	Zr	8.088	Hf	8.099
Cr	8.029	Nb	8.037	Ta	8.044
Mn	8.063	Mo	8.028	W	8.018
Fe	8.040	Tc	8.009	Re	8.008
Co	8.042	Ru	8.009	Os	8.006
Ni	8.036	Rh	8.003	Ir	8.000
Cu	8.064	Pd	8.023	Au	8.046

Table S4. Pt-Pt distances ($d_{Pt\text{-}Pt}$, in Å) and Pt-M distances ($d_{Pt\text{-}M}$, in Å) for (111) surfaces Pt_3M of alloying with various guest metals. The Pt-Pt distance for the pure Pt(111) surface is 2.81 Å.

Element	$d_{Pt\text{-}Pt}$	$d_{Pt\text{-}M}$	Element	$d_{Pt\text{-}Pt}$	$d_{Pt\text{-}M}$	Element	$d_{Pt\text{-}Pt}$	$d_{Pt\text{-}M}$
Sc	2.88	2.83	Zn	2.80	2.79	Ag	2.81	2.84
Ti	2.85	2.80	Y	2.95	2.92	La	2.98	3.02
V	2.84	2.77	Zr	2.92	2.86	Hf	2.81	2.85
Cr	2.82	2.77	Nb	2.85	2.83	Ta	2.85	2.83
Mn	2.74	2.71	Mo	2.85	2.81	W	2.85	2.81
Fe	2.81	2.77	Tc	2.82	2.79	Re	2.83	2.80
Co	2.78	2.75	Ru	2.81	2.78	Os	2.81	2.79
Ni	2.77	2.75	Rh	2.77	2.79	Ir	2.82	2.80
Cu	2.78	2.76	Pd	2.79	2.80	Au	2.79	2.85

Table S5. Surface d-band fillings of d-block elements in a Fm-3m structure.

Element	Surface	Free	Element	Surface	Free
Sc	1.09	1	Tc	6.59	5
Ti	1.99	2	Ru	5.97	7
V	3.09	3	Rh	7.11	8
Cr	4.23	5	Pd	8.51	10
Mn	5.22	5	Ag	9.18	10
Fe	6.23	6	La	0.97	1
Co	7.27	7	Hf	1.62	2
Ni	8.35	8	Ta	2.32	3
Cu	9.21	10	W	3.33	4
Zn	9.73	10	Re	5.07	5
Y	1.00	1	Os	6.03	6
Zr	1.71	2	Ir	6.95	7
Nb	2.51	4	Pt	8.02	9
Mo	3.94	5	Au	8.77	10

References

(1) Liu, W.; Ruiz, V. G.; Zhang, G.-X.; Santra, B.; Ren, X.; Scheffler, M.; Tkatchenko, A. Structure and energetics of benzene adsorbed on transition-metal surfaces: density-functional theory with van der Waals interactions including collective substrate response. *New J. Phys.* **2013**, *15*, 053046.

(2) Peng, H.; Yang, Z.-H.; Perdew, J. P.; Sun, J. Versatile van der Waals Density Functional Based on a Meta-Generalized Gradient Approximation. *Phys. Rev. X* **2016**, *6*, 041005.

(3) Harrison, W. A.; Froyen, S. Universal linear-combination-of-atomic-orbitals parameters for d-state solids. *Phys. Rev. B* **1980**, *21*, 3214.

(4) Kitchin, J. R.; Nørskov, J. K.; Barteau, M. A.; Chen, J. G. Modification of the surface electronic and chemical properties of Pt(111) by subsurface 3d transition metals. *J. Chem. Phys.* **2004**, *120*, 10240.

(5) Borca, B.; Schendel, V.; Petuya, R.; Pentegov, I.; Michnowicz, T.; Kraft, U.; Klauk, H.; Arnau, A.; Wahl, P.; Schlickum, U.; Kern, K. Bipolar Conductance Switching of Single Anthradithiophene Molecules. *ACS Nano* **2015**, *9*, 12506.

(6) Binnig, G.; Rohrer, H.; Gerber, C.; Weibel, E. Surface Studies by Scanning Tunneling Microscopy. *Phys. Rev. Lett.* **1982**, *49*, 57.

(7) Li, J.; Hou, S.; Yao, Y.-R.; Zhang, C.; Wu, Q.; Wang, H.-C.; Zhang, H.; Liu, X.; Tang, C.; Wei, M.; Xu, W.; Wang, Y.; Zheng, J.; Pan, Z.; Kang, L.; Liu, J.; Shi, J.; Yang, Y.; Lambert, C. J.; Xie, S.-Y.; Hong, W. Room-temperature logic-in-memory operations in single-metallofullerene devices. *Nat. Mater.* **2022**, *21*, 917.

(8) Haynes, W. M. *CRC Handbook of Chemistry and Physics*; CRCPress: Boca Raton, Florida, 2016.

(9) Tantardini, C.; Oganov, A. R. Thermochemical electronegativities of the elements. *Nat. Commun.* **2021**, *12*, 2087.

(10) Jackson, M.; BarChart Inc: Boca Raton, 2015.

(11) Cordero, B.; Gómez, V.; Platero-Prats, A. E.; Revés, M.; Echeverría, J.; Cremades, E.; Barragán, F.; Alvarez, S. Covalent radii revisited. *Dalton Transactions* **2008**, 2832.

(12) Waber, J. T.; Cromer, D. T. Orbital Radii of Atoms and Ions. *J. Chem. Phys.* **1965**, *42*, 4116.
